# Structure-function and rational design of a spider toxin Ssp1a at human voltage-gated sodium channel subtypes

**DOI:** 10.3389/fphar.2023.1277143

**Published:** 2023-11-13

**Authors:** Yashad Dongol, David T. Wilson, Norelle L. Daly, Fernanda C. Cardoso, Richard J. Lewis

**Affiliations:** ^1^ Centre for Chemistry and Drug Discovery, Institute for Molecular Bioscience, The University of Queensland, Brisbane, QLD, Australia; ^2^ Australian Institute of Tropical Health and Medicine, James Cook University, Cairns, QLD, Australia

**Keywords:** ICK toxins, rational design, spider toxin, Ssp1a, structure-function, voltage-gated sodium channels

## Abstract

The structure-function and optimization studies of Na_V_-inhibiting spider toxins have focused on developing selective inhibitors for peripheral pain-sensing Na_V_1.7. With several Na_V_ subtypes emerging as potential therapeutic targets, structure-function analysis of Na_V_-inhibiting spider toxins at such subtypes is warranted. Using the recently discovered spider toxin Ssp1a, this study extends the structure-function relationships of Na_V_-inhibiting spider toxins beyond Na_V_1.7 to include the epilepsy target Na_V_1.2 and the pain target Na_V_1.3. Based on these results and docking studies, we designed analogues for improved potency and/or subtype-selectivity, with S7R-E18K-rSsp1a and N14D-P27R-rSsp1a identified as promising leads. S7R-E18K-rSsp1a increased the rSsp1a potency at these three Na_V_ subtypes, especially at Na_V_1.3 (∼10-fold), while N14D-P27R-rSsp1a enhanced Na_V_1.2/1.7 selectivity over Na_V_1.3. This study highlights the challenge of developing subtype-selective spider toxin inhibitors across multiple Na_V_ subtypes that might offer a more effective therapeutic approach. The findings of this study provide a basis for further rational design of Ssp1a and related NaSpTx1 homologs targeting Na_V_1.2, Na_V_1.3 and/or Na_V_1.7 as research tools and therapeutic leads.

## 1 Introduction

Voltage-gated sodium (Na_V_) channels underpin electrical signaling by allowing passive and rapid influx of Na^+^ ions necessary to control initiation and propagation of action potentials in electrically excitable cells, including neurons and muscles (Ahern et al., 2016). Accordingly, Na_V_ channel dysfunction is associated with various neuronal and neuromuscular disorders, including pain, epilepsy, arrythmia and myopathy (de Lera Ruiz and Kraus, 2015; Cardoso and Lewis, 2018; Dib-Hajj and Waxman, 2019; Cardoso, 2020; Menezes et al., 2020; Goodwin and McMahon, 2021). These channels can be pharmacologically modulated by neurotoxins that bind to different sites on the Na_V_ channel to alter the voltage-dependence of activation, inactivation, and conductance (Stevens et al., 2011; de Lera Ruiz and Kraus, 2015). Peptidic gating modifier toxins, including spider toxins, preferentially target the extracellular binding sites located in the domain II (DII) and domain IV (DIV) of the Na_V_ channels to modulate the channel gating. The Na_V_ channel is structurally composed of four non-homologous domains (DI–DIV), which collectively forms the functional, pore-forming α-subunit (Shen et al., 2017; Dongol et al., 2019). There are nine known human Na_V_ isoforms (hNa_V_1.1–1.9), each with distinct tissue localization, channel kinetics and physiological functions (de Lera Ruiz and Kraus, 2015). Modulating a specific isoform is key to avoiding side effects associated with the use of Na_V_-inhibitor drugs; however, the high structural homology between Na_V_ subtypes (Vetter et al., 2017) remains a challenge in obtaining subtype-selective inhibitors, which could be addressed by optimizing ligands, including venom peptides. Venoms evolved for prey capture and/or defense are rich in peptide Na_V_-modulators (Kalia et al., 2015). Research in the 1980s first identified the Na_V_-modulating effects of spider venom toxins (Fontana and Vital-Brazil, 1985; Adams et al., 1989), many of which are now used as research tools to help define the structure, function and pharmacology of Na_V_ channels (Escoubas et al., 2000; Stevens et al., 2011; Kalia et al., 2015; Wu et al., 2018) and their role in disease (Osteen et al., 2016). More recently, these complex venom peptide libraries have been exploited for potential drug leads (Saez et al., 2010; Pineda et al., 2014; Cardoso and Lewis, 2019; Saez and Herzig, 2019; Cardoso et al., 2022), including CcoTx-1 (Shcherbatko et al., 2016), GpTx-1 (Murray et al., 2015; Murray et al., 2016), ProTx-II (Flinspach et al., 2017) and Tap1a (Hu et al., 2021).

**FIGURE 1:**
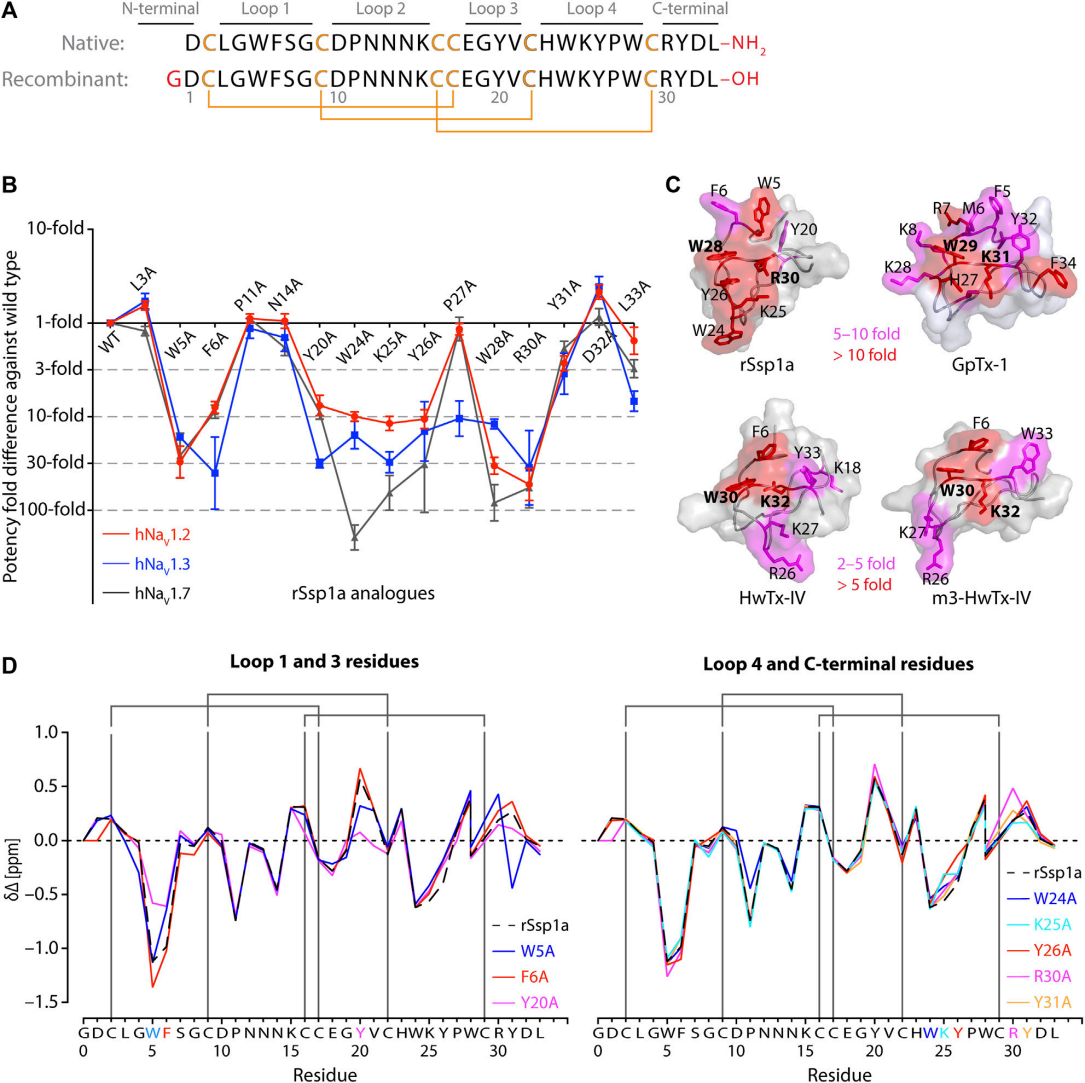
Structure-function of Ssp1a. **(A)** Primary structure of native Ssp1a and recombinant Ssp1a (rSsp1a) showing the disulfide bond connectivity. **(B)** Fold potency difference of rSsp1a alanine mutants vs. wild type (WT) rSsp1a at hNa_V_1.2, hNa_V_1.3 and hNa_V_1.7. Data were presented as means ± SEM, with *n* = 3–12. The W5A, F6A, W24A, K25A, Y26A, W28A, R30A did not complete the dose-response curve at hNa_V_1.2, hNa_V_1.3, and hNa_V_1.7 at the maximum concentration tested. Similarly, Y20A, P27A, Y31A and L33A at hNa_V_1.3 also demonstrated incomplete dose-response. The IC_50_ values from the incomplete dose-response for these mutants were used to plot the panel for better comparison of rSsp1a residues key to hNa_V_1.2, hNa_V_1.3, and hNa_V_1.7 activity. The readers are requested to follow Supplementary Table S1 for relative IC_50_ values and Supplementary Figures S1 for dose-response curves. **(C)** Active residues in the surface of rSsp1a (PDB: 7SKC) Dongol et al., 2021), GpTx-1 (modeled on engineered GPTX-1, PDB: 6MK5) (Murray et al., 2015), HwTx-IV (PDB: 2M4X) Revell et al., 2013) and m3-HwTx-IV (PDB: 5T3M) (Wisedchaisri et al., 2021) were compared to identify which alanine mutation reduced the toxin potency at hNa_V_1.7 as indicated by the respective color code (red or pink). All four toxins were aligned at NaSpTx1 signature motif WCK/R (W and K/R bolded). **(D)** Secondary Hα chemical shift of rSsp1a and the alanine mutants. The secondary shifts were derived by subtracting the random coil Hα shift from the experimental Hα shifts for the eight alanine substitutions distributed across loop 1, loop 3, loop 4 and the C-terminal. The Hα for rSsp1a shifted in W5A, F6A and Y20A mutations.

Ssp1a, a 33-residue inhibitor cystine knot (ICK) peptide (Figure 1A) belonging to the voltage-gated sodium channel modulator spider toxin family 1 (NaSpTx1), is a potent inhibitor of neuronal hNa_V_ subtypes 1.7, 1.6, 1.3, 1.2 and 1.1 (Dongol et al., 2021). The closest homologs with comprehensive structure-function data available are the distantly related GpTx-1 (44% identity) and HwTx-IV (40% identity), with HwTx-IV and recombinant Ssp1a (rSsp1a) showing similar pharmacology at hNa_V_1.7 (Xiao et al., 2008; Dongol et al., 2021). While the NaSpTx1 toxin studies have focused on the development of hNa_V_1.7-selective inhibitors (Minassian et al., 2013; Revell et al., 2013; Klint et al., 2015; Murray et al., 2015; Murray et al., 2016; Shcherbatko et al., 2016; Rahnama et al., 2017; Zhang et al., 2018; Neff et al., 2020), the determinants of NaSpTx1 pharmacology at the potential pain target hNa_V_1.3 (Black et al., 1999; Kim et al., 2001; Hains et al., 2004; Hong et al., 2004; Garry et al., 2005; Lindia et al., 2005; Black et al., 2008; Chen et al., 2014; Tan et al., 2015; Xu et al., 2016) and epilepsy target hNa_V_1.2 (Menezes et al., 2020) have been largely ignored. Therefore, the characterization of Ssp1a in this study could provide common structure-function information for several closer uncharacterized homologs and at targets hNa_V_1.2 and hNa_V_1.3, in addition to hNa_V_1.7.

In this study, 15 alanine mutants of rSsp1a in the Na_V_ pharmacophore region of NaSpTx1 inhibitors (Li et al., 2004; Klint et al., 2012; Minassian et al., 2013; Revell et al., 2013; Murray et al., 2015; Murray et al., 2016; Shcherbatko et al., 2016) identified the Ssp1a-specific pharmacophore. Using these restraints, docking studies identified specific molecular interactions between rSsp1a and hNa_V_1.2, hNa_V_1.3 and hNa_V_1.7. Through structure-function studies of rSsp1a activity at hNa_V_1.2, hNa_V_1.3 and hNa_V_1.7 and previous optimization studies of NaSpTx1 peptides (Minassian et al., 2013; Revell et al., 2013; Murray et al., 2015; Zhang et al., 2015; Murray et al., 2016; Shcherbatko et al., 2016; Zhang et al., 2018; Neff et al., 2020), we designed rSsp1a analogues with significantly improved potency and subtype-selectivity for hNa_V_1.3 and hNa_V_1.2/hNa_V_1.7, respectively. The findings of this study provide insight into rational design of rSsp1a and NaSpTx1 homologs targeting the hNa_V_1.2, hNa_V_1.3, and hNa_V_1.7 subtypes, with single or multiple subtype-selectivity, with the aim of developing lead molecules that have high value as research tools and/or therapeutic agents.

## 2 Materials and methods

### 2.1 Cell culture

Human embryonic kidney 293 (HEK293) cells stably expressing recombinant hNa_V_1.2, hNa_V_1.3 and hNa_V_1.7 and the β1 auxiliary subunit (Scottish Biomedical Drug Discovery, Glasgow, UK) were cultured in Minimal Essential medium (MEM) (Sigma-Aldrich, MO, United States) supplemented with 10% v/v FBS-New Zealand origin (Assay Matrix), 2 mM L-glutamine and selection antibiotics as per manufacturer’s recommendation. The HEK293 cells heterologously expressing mNa_V_1.7 and F813G-mNa_V_1.7 were generously provided by Prof Irina Vetter and were cultured in Minimal Essential medium (MEM) (Sigma-Aldrich, MO, United States) supplemented with 10% v/v FBS-New Zealand origin (Assay Matrix), 2 mM L-glutamine and hygromycin 100 μg/mL.

### 2.2 Automated whole-cell patch-clamp electrophysiology

Na_V_ channel currents from HEK293 cells stably expressing Na_V_ subtypes and the β1 auxiliary subunit were recorded using an automated whole-cell patch clamp system QPatch 16X (Sophion Bioscience A/S, Ballerup, Denmark). As per the manufacturer’s guidelines, the cells were cultured for 48 h to achieve ∼80% confluency, detached using Detachin (Genlantis) and resuspended to 5 × 10^6^ cells/mL in serum free media [CHO-cell SFM (Life Technologies), 25 mM HEPES and 100 U/mL penicillin/streptomycin]. The extracellular solution comprised (in mM) 1 CaCl_2_, 1 MgCl_2_, 5 HEPES, 3 KCl, 140 NaCl and 20 TEA-Cl, with the pH adjusted to 7.3 with NaOH. The intracellular solution comprised (in mM) 140 CsF, 1 EGTA, 5 CsOH, 10 HEPES and 10 NaCl, with the pH adjusted to 7.3 with CsOH. The osmolarity of both solutions were adjusted to 320 mOsm with sucrose. Compounds were prepared in extracellular solution containing 0.1% bovine serum albumin (Sigma-Aldrich). To obtain the dose-response curves, cells were maintained at a holding potential −80 mV and Na^+^ currents were elicited by 20 m voltage steps to 0 mV from a −120 mV conditioning pulse applied for 200 m. Increasing concentrations of the peptide were incubated with the cells at the holding potential for 2 min before the voltage protocol was applied.

### 2.3 Nuclear magnetic resonance (NMR) structure determination of rSsp1a alanine mutants

Lyophilized peptide (500–1,000 µg) was resuspended in 90% H_2_O:10%D_2_O. Two-dimensional ^1^H-^1^H TOCSY and ^1^H-^1^H NOESY spectra were acquired at 290 K using a 600 MHz AVANCE III NMR spectrometer (Bruker, Karlsruhe, Germany) equipped with a cryogenically cooled probe. All spectra were recorded with an interscan delay of 1 s. NOESY spectra were acquired with mixing times of 200–250 ms and TOCSY spectra were acquired with isotropic mixing periods of 80 ms. Two-dimensional spectra were collected over 4,096 data points in the f2 dimension and 512 increments in the f1 dimension over a spectral width of 12 ppm. Standard Bruker pulse sequences were used with an excitation sculpting scheme for solvent suppression. NMR assignments were made using established protocols (Wüthrich, 1983), and the secondary shifts derived by subtracting the random coil αH shift from the experimental αH shifts (Wishart et al., 1995). Spectra were recorded referenced to external 4,4-dimethyl-4-silapentane-1-sulfonic acid (DSS).

### 2.4 Alanine scanning and rational design of rSsp1a

Based on homology and earlier structure-function studies of NaSpTx1 toxins, 16 residues were selected for alanine substitution. The rSsp1a structure-function data across hNa_V_1.2, hNa_V_1.3, and hNa_V_1.7 were then used along with the homology and structure-function information of other NaSpTx1 toxins to guide rational design of rSsp1a. Unfortunately, the docking model of rSsp1a at hNa_V_ subtypes were not used for rSsp1a rational design as the resting structure of hNa_V_1.7 DII was published (Wisedchaisri et al., 2021) after the mutants were made. In total, sixteen positions in rSsp1a were mutated to recombinantly produce 38 rSsp1a-analogues, including 30 single mutants, five double mutants, one mutant with an N-terminal extension and two mutants with a C-terminal extension. A N-terminal extended version (GP-Ssp1a) was designed to mimic GP-HwTx-IV (Neff et al., 2020) and GP-ProTx-II (Flinspach et al., 2017), whereas a C-terminal extension (rSsp1a-GK) was designed to mimic the C-terminal amide version of HwTx-IV (Minassian et al., 2013; Neff et al., 2020). Double mutants were designed based on the activity of each single mutation at the three hNa_V_ subtypes.

The rSsp1a mutant plasmids were produced using a QuikChange Lightning Site-Directed Mutagenesis Kit (Agilent Technologies) and Ssp1a-pLicC plasmid construct (GeneArt Gene Synthesis, Life Technologies) comprising MalE signal sequence for periplasmic export, His_6_ affinity tag, maltose binding protein (MBP) tag, a tobacco etch virus (TEV) recognition and cleavage sequence. Briefly, primers were designed using SnapGene software and purchased from Sigma-Aldrich, and the mutant strand was synthesized using polymerase chain reaction (PCR). The amplified PCR product was digested with Dpn I restriction enzyme to eliminate the parental rSsp1a construct and was transformed into Competent *E. coli* TOP10 cells. The transformed TOP10 cells were plated on Luria-Bertani (LB) agar plates containing 100 μg/mL ampicillin (Amp) and incubated overnight at 37°C. The isolated colonies were sub-cultured overnight at 37°C at 120 rpm, and the plasmid DNA was extracted using a QIAprep Spin Miniprep kit (QIAGEN) following manufacturer’s protocol. The desired mutation in the extracted DNA was confirmed by Sanger sequencing at the Australian Genome Research Facility, Brisbane using Big Dye Terminator (BDT) chemistry version 3.1 (Applied Biosystem). Double mutant plasmids were made using rSsp1a single mutant plasmids as a base construct and a set of primers for the second mutation, and following site-directed mutagenesis, miniprep and Sanger sequencing as described above.

### 2.5 Recombinant production of rSsp1a analogues

rSsp1a analogues were recombinantly expressed in *E. coli* as a His_6_ tagged-MBP fused peptide, harvested, and purified as described previously (Dongol et al., 2021). Briefly, the mutant plasmids were transformed into BL21 (λDE3) competent *E. coli* cells and cultured in LB-Amp medium at 37°C, 120 rpm until the optical density at 600 nm (OD600) reached 0.8–1.0. Peptide expression was induced with 500 μM isopropyl β-D-1-thiogalactopyranoside (IPTG) at 16°C and 120 rpm overnight and pelleted at 6,000 rpm for 10 min at 4°C. The pellet was resuspended in TN buffer (Tris 25 mM, NaCl 150 mM, pH 8.0) and lysed in a constant pressure cell disruptor at 25 kPa at 4°C–8°C. The fusion protein contained in the cell lysate was captured by passing the lysate supernatant through Ni-NTA resin (Hispur NiNTA, Thermo Scientific) and then eluted with TN buffer containing 500 mM imidazole. After desalting, the fusion protein was cleaved with TEV protease in the reducing environment provided by glutathione redox pair. The post-cleavage sample was filtered through a centrifuge filter to isolate the cleaved peptide from tag proteins and loaded onto a reversed-phase C_18_ column (30Å, 5μm, 4.6 × 250mm, Vydac 218TP, Grace) on an Agilent 1100 series HPLC for purification. The peptide was eluted using the following gradient of solvent B (90% ACN, 0.05% TFA in MilliQ water) in solvent A (0.05% TFA in MilliQ water): 5% solvent B over 0–5 min, 5%–10% solvent B over 5–10 min, 10%–50% solvent B over 10–40 min, 50%–80% solvent B over 40–45 min, wash at 80% solvent B over 45–50 min, 80%–5% solvent B over 50–55 min and a final wash with 5% solvent B over 55–65 min at a flow rate of 1 mL/min. Peak fractions at 214 nm were collected, checked the purity, analysed for the mass using MALDI-TOF/TOF (SCIEX 5800), lyophilized, quantitated using nanodrop, and stored at −20°C until use.

### 2.6 Molecular docking

The recently solved structure of Na_V_Ab/Na_V_1.7 VS2A chimera (PDB: 7K48) (Wisedchaisri et al., 2021) provided the resting state structure of the Na_V_1.7 DII, which was used as a template to build a homology model for hNa_V_1.2 DII (UniProt: Q99250) and hNa_V_1.3 DII (UniProt: Q9NY46) in the resting state using SWISS-MODEL (Waterhouse et al., 2018). HADDOCK2.2 webserver was used to perform data-driven docking studies under the Easy interface mode, which only requires the starting structures and the restraint definitions in the form of active and passive residues to drive the docking (de Vries et al., 2010; Van Zundert et al., 2016). Our NMR structure of rSsp1a, cryo-EM structure of the Na_V_Ab/Na_V_1.7 VS2A chimera, and homology models of DII of hNa_V_1.2 and hNa_V_1.3 were uploaded with the structure and restraint definitions to generate putative ligand receptor complex. The rSsp1a active residues were defined based on our activity data of rSsp1a alanine mutants at hNa_V_1.2, hNa_V_1.3, and hNa_V_1.7, whereas the active residues on DII of hNa_V_ subtypes were defined based on the previously published channel mutation data (Xiao et al., 2010; Xiao et al., 2011; Cai et al., 2015; Zeng et al., 2018; Xu et al., 2019). The docking program was allowed to define the passive residues automatically around the active residues.

For the rSsp1a–hNa_V_1.2 docking, the rSsp1a active residues defined were W5, F6, W24, K25, Y26, W28, and R30. Similarly, the active residues in hNa_V_1.2 DII S1–S2 loop were E779, Y781, T784, E785, F787, S788 whereas the active residues in the DII S3–S4 loop were E837, E844, and S847. Likewise, the rSsp1a–hNa_V_1.3 docking was driven by defining the rSsp1a active residues W5, F6, Y20, W24, K25, Y26, P27, W28, R30 and L33, and hNa_V_1.3 active residues in the DII S1–S2 loop (E780, Y782, T785, E786, F788, and S789) and DII S3–S4 loop (E838, S842, E845, and S848). Finally, to dock rSsp1a at hNa_V_1.7, the active residues defined in rSsp1a were W5, F6, W24, K25, Y26, W28 and R30, while the active residues defined in the hNa_V_1.7 DII S1–S2 loop were E753, E759, E760, F761 and K762, and active residues defined in the DII S3–S4 loop were E811, F813, D816 and E818. The docking results were displayed as a cluster of water-refined models, which were then downloaded and visualized using Pymol 2.4.1 (Schrodinger, 2018). Generally, the top 10 clusters were listed in the order of their HADDOCK score—the top position in the list occupied by the cluster with the lowest HADDOCK score. Further, each cluster contains the top four best scoring structures. A z-score was also determined for each cluster, indicating the number of standard deviations by which the HADDOCK score of a particular cluster differed from the mean score of all clusters. The top clusters in each list providing the lower z-score values are considered more reliable (de Vries et al., 2010; Van Zundert et al., 2016). Additionally, each of the top 10 generated models were evaluated to identify the docking pose that best supported the pharmacology for interactions. The 3D structure of rSsp1a analogues used for docking studies were obtained by introducing the mutation in the rSsp1a structure using Pymol. The docking of rSsp1a analogues were driven by defining the active residues at rSsp1a analogues and hNa_V_ subtypes as described above for rSsp1a docking at each hNa_V_ subtypes. The presence of key molecular interactions was determined by the distance separating the two specified interacting atoms participating in interactions between the toxin and the channel. Electrostatic interactions were categorized as hydrogen bonds (H-bonds) when an electronegative O-atom engaged with a H-atom covalently bonded either to an electronegative O- or N-atom within 2 Å. Conversely, electrostatic interactions were designated as salt bridges between positively charged N-atom (amino group) and the negatively charged O-atom (carboxyl group) within 3.3 Å. Similarly, hydrophobic interactions were defined interactions between hydrophobic residues of the toxin and channel (<5 Å), with the van der Waals radii represented as dots in the structural images. Dots representations were generated using the default parameters of the Pymol program for atom van der Waals radii, dot radius, width, and density. Additionally, aromatic amino acids from the toxin and the channel, located within a 5 Å distance, were considered to form π-π interactions.

### 2.7 Data analysis

The experimental data were analysed using QPatch Assay software v5.6.4 and GraphPad Prism 7.0 using a four-parameter Hill equation [Y = Bottom + (Top—Bottom)/(1 + 10^(Log IC_50_—X)* Hillslope)] to fit concentration response curves by non-linear regression analysis and Student’s t-test. Data are presented as means ± standard error of mean (SEM) with number of independent experiments stated and *p <* 0.05 is considered statistically significant. Statistically insignificant shift in the activity is denoted by “little” while small but statistically significant shift in the activity is denoted by “slight” throughout the manuscript.

## 3 Results

### 3.1 Determining the active surface of rSsp1a

Fifteen rSsp1a alanine-analogues were produced via recombinant expression as previously described (Dongol et al., 2021), and their activity tested on hNa_V_1.2, hNa_V_1.3 and hNa_V_1.7 as a function of varying concentration (Figure 1B; Supplementary Figure S1; Supplementary Table S1). The alanine mutations causing ≥10-fold loss in potency at all three hNa_V_ subtypes included W5A, F6A, W24A, K25A, Y26A, W28A and R30A. Y20A was inactive at hNa_V_1.3 but reduced hNa_V_1.2 and hNa_V_1.7 activity by nearly 10-fold. The P27A, Y31A and L33A mutations also preferentially impacted hNa_V_1.3. Surprisingly, the conserved P11 played little role in rSsp1a activity, and little to slight effects were observed for the N14A mutant. Two mutants, L3A and D32A, slightly improved the rSsp1a potency at hNa_V_1.2, but only D32A slightly enhanced rSsp1a potency at hNa_V_1.3.

We compared the active surface of rSsp1a with GpTx-1, HwTx-IV, and the m3-HwTx-IV optimized variant, which revealed that the hydrophobic and basic residues that comprise the active surface in these toxins are distributed around a central WCK/R motif, with key residues aligned at corresponding positions in the 3D structure (Figure 1C). All active residues were solvent exposed and on the same face of these toxins, except Y20 in rSsp1a which was buried and K18 in HwTx-IV which was located on the opposite face. The loop 1, loop 4 and C-terminal region act in concert to form the active surface of these NaSpTx1 toxins as previously described for the spider peptide Tap1a (Hu et al., 2021).

Next, we determined whether alanine substitutions reducing rSsp1a activity were structural by assessing influences on the Hα secondary chemical shifts of rSsp1a alanine mutants with significant activity loss (Figure 1D). Mutant W5A produced a significant change in Hα chemical shift at Y20 and Y31, while F6A only affected the neighboring W5. The loop 3 mutant Y20A affected both W5 and F6 Hα chemical shifts likely due to their spatial proximity to the buried Y20, suggesting loop 3 residues play a structural role in these ICK peptides. The loop 4 mutations W24A, K25A and Y26A and C-terminal mutations R30A and Y31A had only minor local effects on the Hα chemical shifts, indicating that the activity losses attributed to these mutations are functional rather than structural. Unfortunately, W28A-rSsp1a analogue was obtained in insufficient yield for NMR analysis.

### 3.2 Molecular interaction of rSsp1a at hNa_V_1.2, hNa_V_1.3 and hNa_V_1.7

Our previous work indicated that rSsp1a traps DII of hNa_V_1.7 in the resting conformation (Dongol et al., 2021). The resting state structure of Na_V_1.7-DII trapped by m3-HwTx-IV was recently solved (Wisedchaisri et al., 2021), allowing predictive docking of rSsp1a using HADDOCK (Van Zundert et al., 2016). We validated the HADDOCK docking results by comparing the docking orientation and molecular interaction of m3-HwTx-IV at Na_V_Ab/Na_V_1.7 VS2A between the HADDOCK-generated *in silico* structure and the cryo-EM structure captured by Wisedchaisri et al. (Wisedchaisri et al., 2021) (Supplementary Figure S2).

**FIGURE 2:**
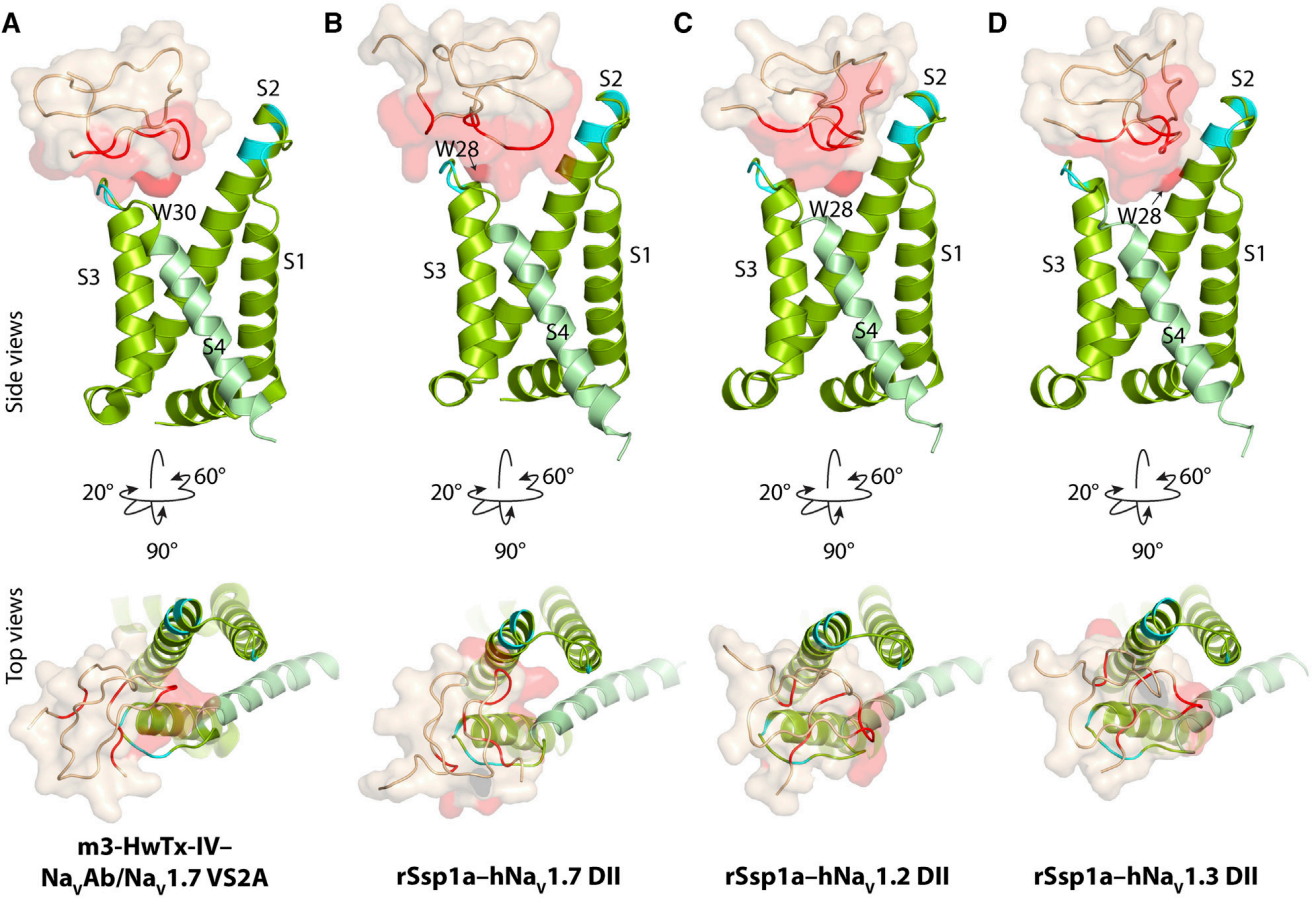
Docking pose of m3-HwTx-IV and rSsp1a. **(A)** Docking orientation of m3-HwTx-IV at Na_V_Ab/Na_V_1.7 VS2A captured in cryo-EM and **(B–D)** docking orientation of rSsp1a at hNa_V_1.2, hNa_V_1.3, and hNa_V_1.7 obtained using HADDOCK are shown with side and top views. The red surface indicates the activity surface of the corresponding toxin, with dark red indicating the Trp residue from the WCK/R motif. The cyan colour indicates non-identical residues among hNa_V_1.2, hNa_V_1.3, and hNa_V_1.7. The recently published Na_V_Ab/Na_V_1.7 VS2A structure (Wisedchaisri et al., 2021) provided the resting structure of DII that served as the hNa_V_1.7 DII used to model the hNa_V_1.2 and hNa_V_1.3 DII resting state.

The docking results of rSsp1a to hNa_V_1.2-, hNa_V_1.3- and hNa_V_1.7-DII illustrated that rSsp1a bound in the aqueous cleft formed between the S1–S2 and S3–S4 loops of each channel subtype, primarily targeting the S3–S4 loop as observed for m3-HwTx-IV (Figures 2A–D). rSsp1a docked at hNa_V_1.7 similar to m3-HwTx-IV, with the key residue W28 (W30 in m3-HwTx-IV, Supplementary Figures S3A) positioned towards the S3–S4 loop in the aqueous cleft (Figures 2A, B) and interacting with hydrophobic residues in the S3–S4 loop to trap the S4 segment, as shown for m3-HwTx-IV in Supplementary Figures S3B. When compared to hNa_V_1.7, the docking orientation of rSsp1a at hNa_V_1.2 and hNa_V_1.3 twisted, which allowed rSsp1a to occupy more space in the DII aqueous cleft with W28 occupying the center of the cleft (Figures 2C, D).

**FIGURE 3:**
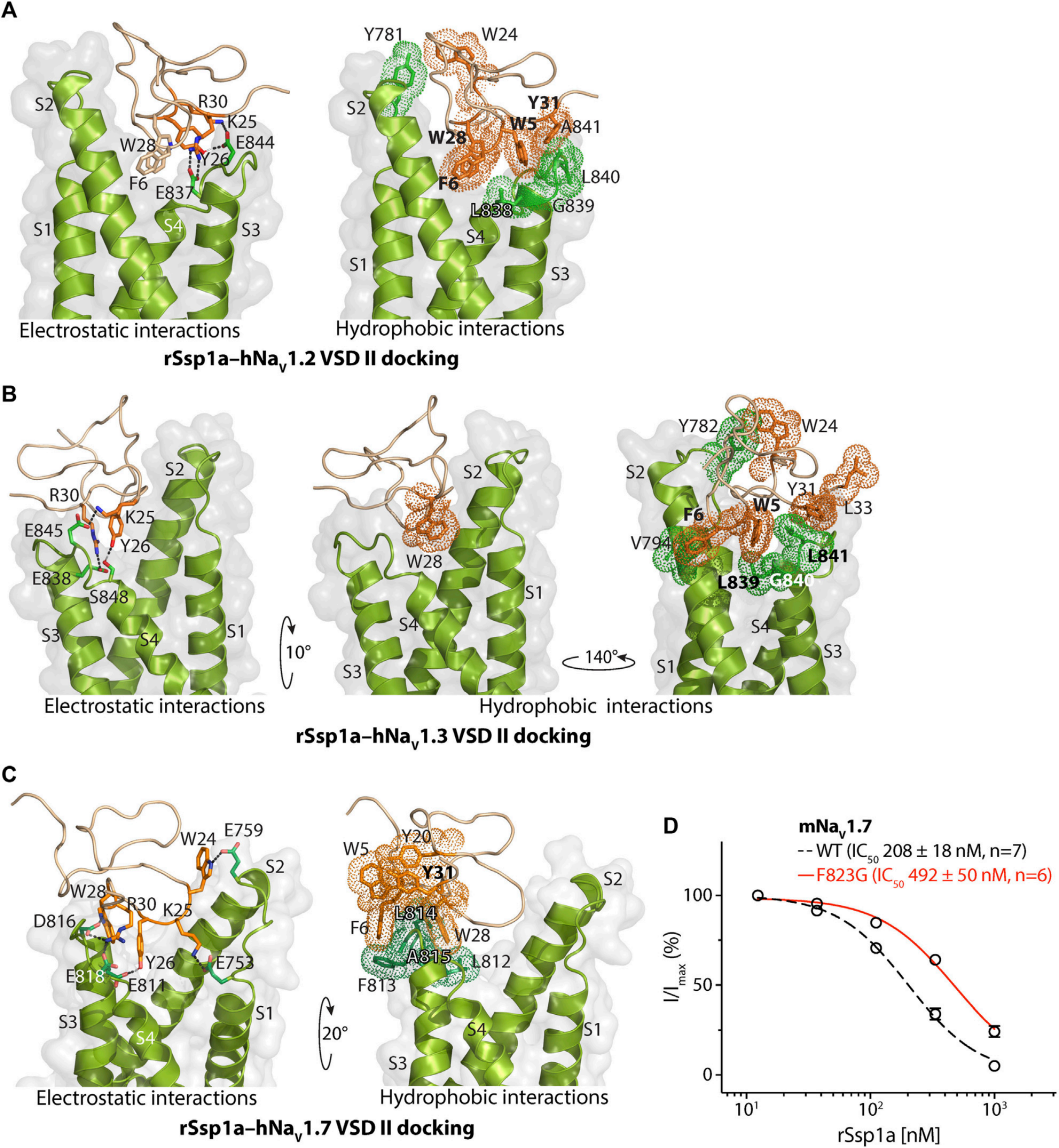
Molecular docking of rSsp1a. Molecular docking of rSsp1a at **(A)** hNa_V_1.2 DII **(B)** hNa_V_1.3 DII and **(C)** hNa_V_1.3 DII illustrating electrostatic and hydrophobic interactions. The resting conformation of hNa_V_1.2 and hNa_V_1.3 DII was modelled on the resting state structure of Na_V_Ab/Na_V_1.7-VS2A chimera (PDB: 7K48) (Wisedchaisri et al., 2021). HADDOCK2.2 Easy interface (Van Zundert et al., 2016) was used for docking and the results were visualized using Pymol 2.4.1 (Schrodinger, 2018). The green and orange dots around the residues corresponds to the van der Waals radii. **(D)** Dose-response of rSsp1a at wild type (WT) and F823G-mNa_V_1.7. Data presented as means ± SEM, with number of experiments (n) indicated in the legend.

The docking orientation of rSsp1a at hNa_V_1.2 revealed molecular interactions, including salt bridges, H-bonds and a series of hydrophobic interactions within the aqueous cleft (Figure 3A). Specifically, K25–E844 (2.8 Å, -NH_3_^+^••••^−^OOC-) and R30–E837 (2.6 Å, =NH_2_^+^••••^−^OOC-) salt bridges, and a H-bond between Y26 and E844 (1.8 Å, -OH••••^−^OOC-) were observed. Additionally, W5 projects into the hydrophobic pocket formed by the LGLA residues in the DII S3–S4 loop and Y31 interacted with W5–LGLA complex, while F6 and W28 occupied the aqueous cleft and interacted with the neighboring hydrophobic residues in the DII S2 and S3–S4 loop. Further, an interaction was observed between W24, located on the edge of the hydrophobic patch in the rSsp1a, and Y781 from the DII S1–S2 loop.

Similar to the rSsp1a–hNa_V_1.2 results, the rSsp1a–hNa_V_1.3 docking orientation also showed K25 and R30 forming a salt bridge with E845 (3.0 Å, -NH_3_^+^••••^−^OOC-) and E838 (2.6 Å, =NH_2_^+^••••^−^OOC-), respectively, and Y26 forming a H-bond with S848 (1.7 Å, -O••••HO-) (Figure 3B). The bulky hydrophobic residue W5 fit within the hydrophobic pocket formed by LGL in the DII S3–S4 loop, with Y31and L33 positioned above the W5–LGL complex to hinder the upward transition of the S4, while F6 and V794 from S2 segment formed a hydrophobic interaction. The toxin penetrated deep into the channel where the bulky hydrophobic W28 occupied the aqueous cleft and interacted with the neighboring hydrophobic residues from DII S1 and S2 segments. Consistent with the hNa_V_1.2 docking, the uniquely positioned W24 showed a hydrophobic interaction with Y782 in the DII S1–S2 loop to further stabilize rSsp1a interactions at the S3–S4 loop.

rSsp1a docked to hNa_V_1.7 DII in a slightly altered orientation compared to hNa_V_1.2 and hNa_V_1.3. Salt bridges between K25–E753 (2.7 Å, -NH_3_^+^••••^−^OOC-), R30–E818 (2.6 Å, =NH_2_^+^••••^−^OOC-) and R30–D816 (2.7 Å, =NH_2_^+^••••^−^OOC-) were observed, with additional electrostatic interactions between W24–E759 (1.8 Å, >NH••••^−^OOC-), W28–D816 (1.8 Å, >NH••••^−^OOC-) and Y26–E810 (1.8 Å, -OH••••^−^OOC-) (Figure 3C). The hydrophobic residues LFLA in the S3–S4 loop project into the hydrophobic groove in rSsp1a formed by W5, F6, Y20, Y31, and W28. This binding mode shifted W28 away from the aqueous cleft center towards the S3–S4 loop, with K25 expected to play a key role in restricting the upward movement of S4 upon depolarization to trap DII in the resting state. We also assessed the role of the non-conserved residue F813 from hNa_V_1.7 DII S3–S4 in rSsp1a binding by using a F823G-mNa_V_1.7 variant. The F823 in mNa_V_1.7 DII corresponds to the F813 in the hNa_V_1.7 DII. The F823G mutation slightly decreased the mNa_V_1.7 sensitivity to rSsp1a (∼2.5-fold) but was not critical for rSsp1a interaction (Figure 3D). Channel residues predicted by docking studies to interact with rSsp1a were highlighted in Supplementary Figure S4.

### 3.3 Optimizing rSsp1a

#### 3.3.1 rSsp1a optimization through single residue substitution

**FIGURE 4:**
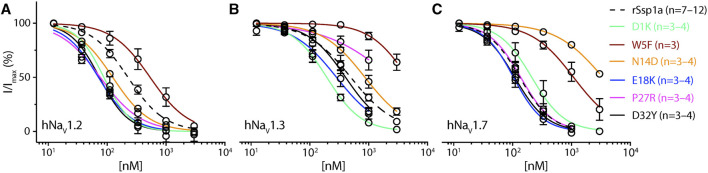
Dose-response of rSsp1a mutations. (**A–C**) Dose-response curve of selected rSsp1a analogues compared with rSsp1a at hNa_V_1.2, hNa_V_1.3, and hNa_V_1.7 obtained using the whole-cell automated patch clamp electrophysiology (QPatch 16X) platform. Data presented as means ± SEM, with number of experiments (n) indicated in the legend.

Based on the active surface defined for rSsp1a through the alanine substitutions and evidence from earlier optimization studies of closely related homologs, we designed and tested several single residue substitutions for rSsp1a (Supplementary Figures S5A–E; Supplementary Figures S6A–F). A charge-reversing substitution of D1K, located away from the predicted interaction face (Supplementary Figures S6A), improved rSsp1a-activity at hNa_V_1.2 and hNa_V_1.3, but not at hNa_V_1.7 (Figure 4; Supplementary Table S2). This suggests that the increased affinity of D1K-rSsp1a may be associated with lipid bilayer interactions that could facilitate interaction with hNa_V_1.2 and hNa_V_1.3. Substitution of the loop 1 hydrophobic residue W5 with a less bulky Phe was tolerated at hNa_V_1.2 but not at hNa_V_1.7 and hNa_V_1.3 (Figure 4; Supplementary Table S2). Other substitutions at loop 1 had smaller effects on rSsp1a activity (Supplementary Table S2; Supplementary Figures S5A) and they surround the rSsp1a interaction face (Supplementary Figures S6B).

**FIGURE 5:**
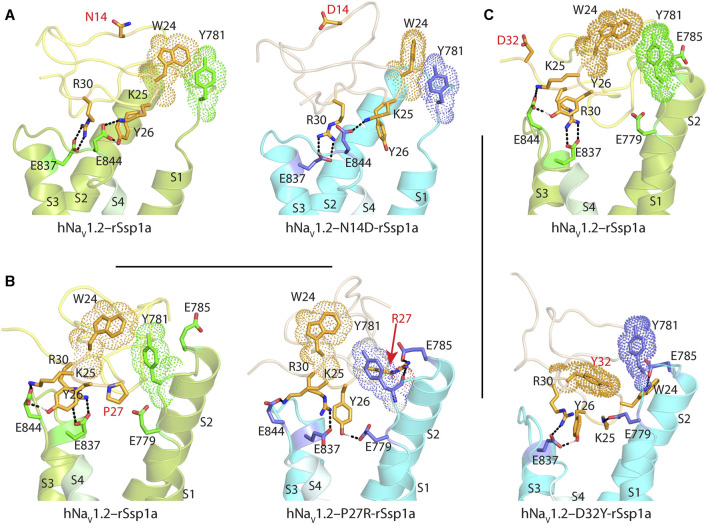
Molecular docking of rSsp1a single mutants. Visualization of molecular interactions of **(A)** N14D-rSsp1a, **(B)** P27R-rSsp1a and **(C)** D32Y-rSsp1a compared to rSsp1a at hNa_V_1.2. Consistent with the activity data, rSsp1a and N14D-rSsp1a docked similarly at hNa_V_1.2 while P27R-rSsp1a formed additional ionic interactions through the substituted R27 and E785 and Y781 (main chain). In addition, Y26–E779 engaged the S1–S2 loop to further strengthen the toxin–channel complex. Compared to rSsp1a, D32Y-rSsp1a formed a stronger interaction with hNa_V_1.2, engaging both the S1–S2 and S3–S4 loops with ionic interactions, and consistent with the activity data.

The surface exposed loop 2 variant N13G-rSsp1a (Supplementary Figures S6C) only slightly weakened activity at hNa_V_1.7 (Supplementary Table S2; Supplementary Figures S5B). In contrast, introduction of an acidic residue Asp at the adjacent residue N14 slightly improved rSsp1a potency at hNa_V_1.2, with little reduction of potency at hNa_V_1.3 and >20-fold reduction of potency at hNa_V_1.7. This provided N14D-rSsp1a variant hNa_V_1.2-selectivity by >24-fold against hNa_V_1.7 and by 7-fold against hNa_V_1.3 (Figure 4; Supplementary Table S2). Reflecting the slight improvement observed at hNa_V_1.2, the docking orientation of N14D-rSsp1a at hNa_V_1.2 (Figure 5A) showed a small shift, which removed the Y26–E844 H-bond but favored a comparatively stronger π-stacking between W24 and Y781. However, in the docking orientation of N14D-rSsp1a at hNa_V_1.7 (Supplementary Figures S7A), the major interaction D816–R30–E818 necessary to trap the DII S4 segment in its resting state was lost, contributing to the weaker toxin-channel interaction observed.

Of the five substitutions made in loop 3 (E18G, E18K, E18F, E18Y and Y20L), only the E18K substitution provided little or slight improvement in rSsp1a potency at all three hNa_V_ subtypes (Figure 4; Supplementary Figures S5B; Supplementary Table S2), with the largest effect observed at hNa_V_1.2 (>3-fold). E18 is spatially distant from the rSsp1a interaction face (Supplementary Figures S6D), suggesting that E18K-rSsp1a might allow longer-range dipole interactions with the lipid bilayer that enhance potency. Y20 is buried within the rSsp1a hydrophobic patch and contributes to rSsp1a activity (Figures 1B, D).

Loop 4 residues (residues 23–28) comprise the active surface of rSsp1a (Supplementary Figures S6E). To examine the role of loop 4, nine analogues mutating five residues from the loop 4 of rSsp1a were generated. Mutations of H23 caused a little increase in rSsp1a activity only for H23S at hNa_V_1.2 (Supplementary Figures S5C; Supplementary Table S2). The W24R mutation partially inhibited hNa_V_1.2 (∼65%), and incompletely inhibited hNa_V_1.7 (∼50%) and hNa_V_1.3 (∼75%) at 3 µM (Supplementary Figures S8). In contrast, the Y26H mutation did not improve rSsp1a activity, while mutations reinstating conserved basic residues at position 27 (P27R and P27K) had opposing affinity at hNa_V_1.3 and hNa_V_1.2, providing >10-fold hNa_V_1.2 selectivity (Figures 4A–C; Supplementary Figures S5C; Supplementary Table S2). However, the P27R and P27K analogues retained rSsp1a activity at hNa_V_1.7 and the fold-potency differences of these two analogues between hNa_V_1.2 and hNa_V_1.7 is <2-fold. We then investigated the molecular basis for the improved selectivity of P27R-rSsp1a between hNa_V_1.2 (Figure 5B) and hNa_V_1.3 (Supplementary Figures S7B). P27R-rSsp1a docked at hNa_V_1.2 in a similar way to rSsp1a but included additional interactions with the S1–S2 loop. Specifically, in addition to the R30–E837 and K25–E844 ionic interactions in the S3–S4 loop, the substituted R27 formed a salt bridge with E785 and a H-bond with Y781 in the main chain, while Y26 interacted with E779 in the S1–S2 loop. These additional interactions at the S1–S2 loop stabilized the toxin-channel complex and improved the potency at hNa_V_1.2. For the P27R-rSsp1a–hNa_V_1.3 complex, the docking orientation most similar to the rSsp1a docking orientation was selected (Supplementary Figures S7B). These results revealed a spatial shift in the P27R-rSsp1a functional residues K25, Y26 and R30 that removed interactions between the active residues K25 and Y26 in rSsp1a and E838 and S848 in hNa_V_1.3. These results are consistent with the reduced activity observed for P27R-rSsp1a at hNa_V_1.3. The W28 residue of the WCK/R motif mutated to generate W28F and W28K analogues had a deleterious effect on hNa_V_ activity; however, the decrease in activity by W28F at hNa_V_1.7 was only ∼5-fold (Supplementary Figures S5C; Supplementary Table S2).

At the C-terminal, we mutated Y31 and D32 to generate six analogues. These two residues are close to the functional residues on the surface of rSsp1a, with Y31 forming the hydrophobic patch (Supplementary Figures S6F). Substitution of Y31 with bulky Trp significantly improved rSsp1a potency, while the Y31T mutation removed the activity (Supplementary Figures S5D; Supplementary Table S2). Substitutions made at D32, preferentially improved rSsp1a potency at hNa_V_1.2 (up to 3-fold) and hNa_V_1.3 (up to 6-fold). The D32F mutant significantly improved rSsp1a potency at hNa_V_1.3 but not selectivity, while the D32Ymutant significantly improved rSsp1a potency (3-fold) and selectivity (∼6-fold) for hNa_V_1.2 against hNa_V_1.3. The docking orientation of D32Y-rSsp1a at hNa_V_1.2 (Figure 5C) differed compared to rSsp1a, allowing engagement of the S1–S2 loop with the ionic bond K25–E779. The DII S3–S4 loop key interactions, including R30–E837 and Y26–E837, were maintained while a new interaction (W24–E785) was formed. In addition, Y32 and Y781 showed the potential to engage through a π–π interaction based on their side chain flexibility; however, the orientation to support a π–π interaction was not evident in the molecular docking studies.

#### 3.3.2 rSsp1a optimization through combinatorial mutation

**FIGURE 6:**
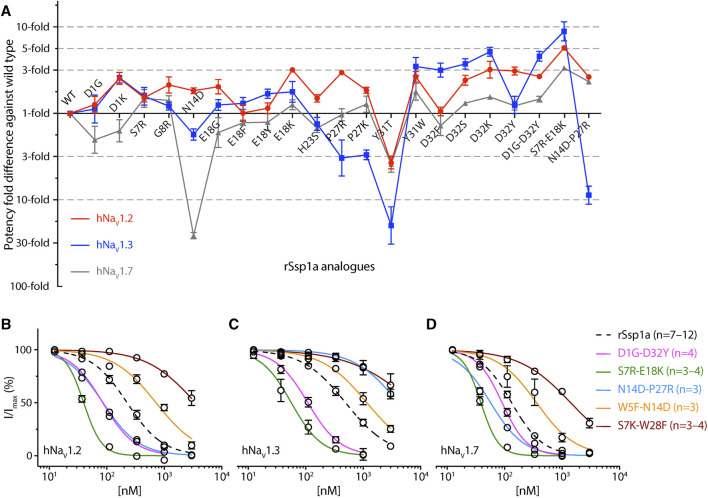
Potency and selectivity optimization of rSsp1a. **(A)** Potency fold-difference of selected rSsp1a analogues presented against the potency of wild type rSsp1a at hNa_V_1.2, hNa_V_1.3, and hNa_V_1.7. Data were presented as means ± SEM (*n* = 3–12, except at hNa_V_1.2 for E18F *n* = 2). **(B–D)** Dose-response curve of rSsp1a double mutants compared with rSsp1a at hNa_V_1.2, hNa_V_1.3 and hNa_V_1.7, obtained using the whole-cell automated patch clamp electrophysiology (QPatch 16X) platform. Data presented as means ± SEM, with number of experiments (n) indicated in the legend.

Guided by the potency and subtype-selectivity results of the single mutants, the double mutants D1G-D32Y, S7R-E18K, N14D-P27R, W5F-N14D and S7K-W28F were synthesised and tested (Figures 6A–D; Table 1). In contrast to the selectivity observed against hNa_V_1.3 for the D32Y mutation, the D1G-D32Y mutation had significantly improved potency at hNa_V_1.3 and hNa_V_1.7 (Figure 6A). The S7R-E18K-rSsp1a analogue showed the greatest improvement in potency of the rSsp1a analogues tested, with hNa_V_1.2 potency enhanced 6-fold, hNa_V_1.3 enhanced ∼10-fold, and hNa_V_1.7 enhanced >3-fold. The N14D-P27R analogue decreased the rSsp1a potency at hNa_V_1.3 (∼40% block at 3µM) and reversed the potency reduction at hNa_V_1.7 caused by the N14D mutation. The results reveal that the potency and subtype-selectivity at hNa_V_1.2 conferred by the N14D and P27R mutations was not additive in the double mutant. The W5F-N14D and S7K-W28F mutants designed for hNa_V_1.2- and hNa_V_1.7-selectivity were unsuccessful.

**TABLE 1:** Double mutant designs for improved subtype-selectivity.

Mutant	Intended subtype-selectivity	Outcome
D1G-D32Y	hNa_V_1.2	Enhanced affinity at hNa_V_1.3 and hNa_V_1.7, generating a non-selective inhibitor with nanomolar potency at the three hNa_V_ subtypes tested
W5F-N14D	hNa_V_1.2	Predicted cumulative effects of single mutants that enhanced hNa_V_1.2 selectivity were not realised
S7R-E18K	hNa_V_1.2 and/or hNa_V_1.3	Enhanced affinity at hNa_V_1.2, hNa_V_1.3 and hNa_V_1.7, generating a non-selective inhibitor with nanomolar potency at the three hNa_V_ subtypes
S7K-W28F	hNa_V_1.7	Reduced potency at hNa_V_1.7
N14D-P27R	hNa_V_1.2	Reverted the potency loss by N14D at hNa_V_1.7 and provided hNa_V_1.2/hNa_V_1.7 selectivity against hNa_V_1.3

**FIGURE 7:**
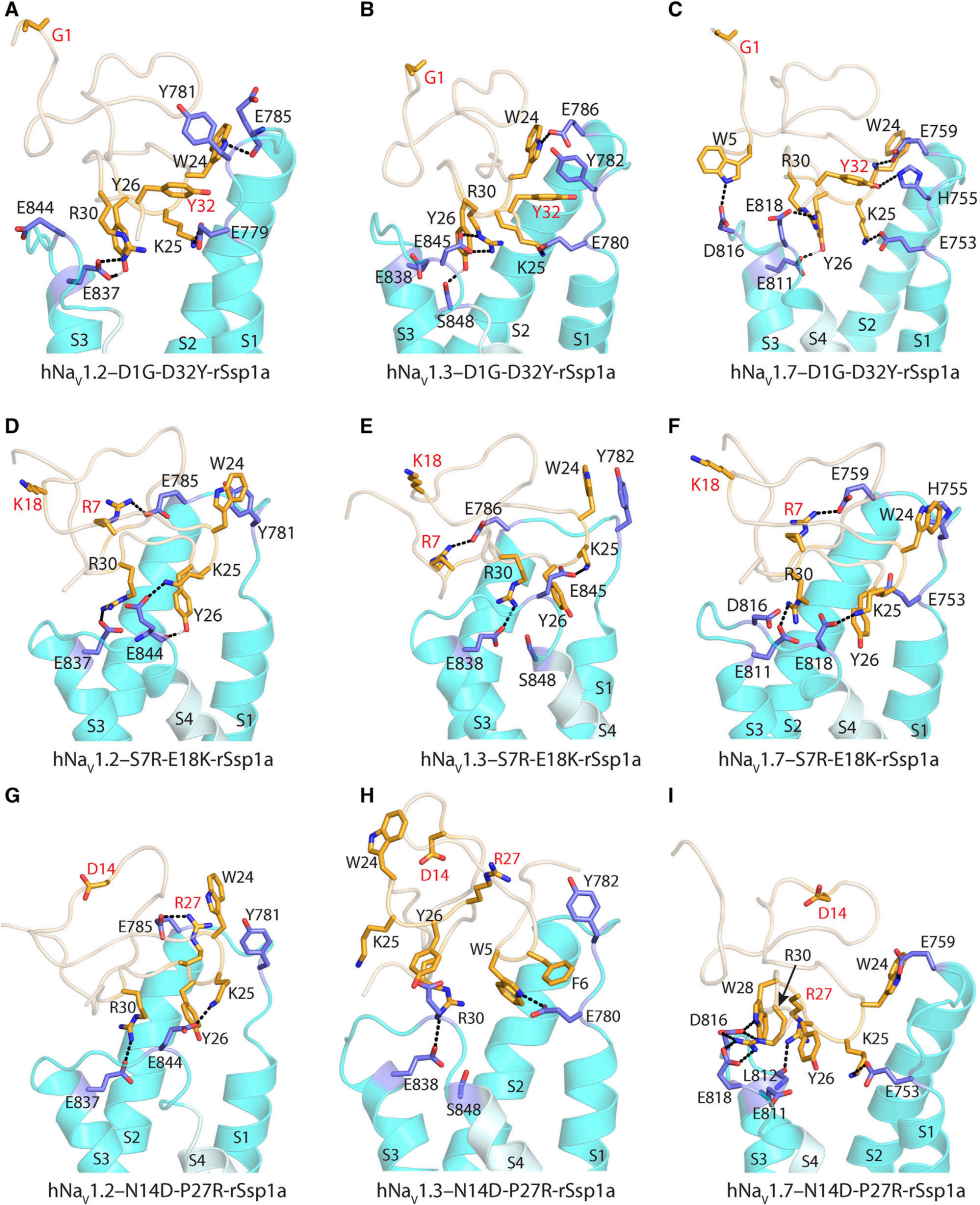
Molecular docking of rSsp1a double mutants. **(A)** D1G-D32Y-rSsp1a docked differently at hNa_V_1.2 compared to rSsp1a (Supplementary Figures S9A) to form stronger ionic bonds, engaging both the S1–S2 and S3–S4 loops, as well as a possible π–π interaction between Y32–Y781, which was consistent with the activity data. **(B)** D1G-D32Y-rSsp1a docked at hNa_**V**_1.3 similar to docking to hNa_**V**_1.2, but in a different orientation compared to rSsp1a (Supplementary Figures S9B), which enabled stronger interactions that engaged both the S1–S2 and S3–S4 loops and a possible Y32–Y782 π–π interaction. **(C)** At hNa_**V**_1.7, both rSsp1a (Supplementary Figures S9C) and D1G-D32Y-rSsp1a docked with a similar orientation, forming new interactions including W5–D816 and Y32–H755. **(D)** At hNa_**V**_1.2, S7R-E18K-rSsp1a docked similar to rSsp1a (Supplementary Figures S9D) with R7 in S7R-E18K-rSsp1a forming an ionic bond with E785 from the S1–S2 loop. In addition, a π–π interaction between W24–Y781 in a parallel displaced geometry was noted. These extra interactions engaging the S1–S2 loop are predicted to form a tighter toxin–channel complex, thus improving the toxin potency. **(E)** S7R-E18K-rSsp1a docked at hNa_V_1.3 similar to docking to hNa_V_1.2 and in an orientation similar to rSsp1a (Supplementary Figures S9E). An additional R7–E786 salt bridge engaging the S1–S2 loop is predicted to form a tighter toxin–channel complex. **(F)** At hNa_V_1.7, S7R-E18K-rSsp1a docked differently to rSsp1a (Supplementary Figures S9F), but contained salt bridges K25–E818, R30–E811 and R7–E759 that tightly engaged the S1–S2 and S3–S4 loop. In addition to the extra R7–E759 salt bridge, a possible W24–H755 π–π interaction was observed. **(G)** At hNa_**V**_1.2, N14D-P27R-rSsp1a docked similar to rSsp1a (Supplementary Figures S9G) with the R27 in N14D-P27R-rSsp1a forming an ionic bond with E785 from the S1–S2 loop. **(H)** The docking orientation for N14D-P27R-rSsp1a at hNa_V_1.3 was chosen to resemble rSsp1a docking. The lack of activity of N14D-P27R-rSsp1a at hNa_V_1.3 was consistent with the absence of major interactions. **(I)** At hNa_V_1.7, N14D-P27R-rSsp1a docked similar to rSsp1a (Supplementary Figures S9I) with R27 interacting with the L812 carbonyl group. The substituted residues are highlighted in red.

Further, docking studies were performed to visualize the molecular basis of potency and subtype-selectivity on the D1G-D32Y-rSsp1a, S7R-E18K-rSsp1a and N14D-P27R-rSsp1a double mutants at the three hNa_V_ subtypes (Figures 7A–I; Supplementary Figures S9A–I). At hNa_V_1.2, D1G-D32Y-rSsp1a docked differently to rSsp1a to form stronger ionic bonds that engaged both the S1–S2 and S3–S4 loops (Figure 7A; Supplementary Figures S9A). The R30–E837 and Y26–E837 interactions engaged the hNa_V_1.2 DII S3–S4 loop, while the K25–E779 and W24–E785 interactions engaged with the S1–S2 loop. The proximity of Y32 to Y781 suggested π–π interactions were probable. Similar interactions were also observed for D1G-D32Y-rSsp1a docking with hNa_V_1.3 (Figure 7B; Supplementary Figures S9B). When docked at hNa_V_1.7, such π–π interaction with Y32 was not formed with the equivalent H755 in the hNa_V_1.7 DII S1–S2 loop. However, interactions between Y32–H755 and W5–D816, in addition to R30–E818, Y26–E811, K25–E753 and W24–E759, predicted enhanced D1G-D32Y-rSsp1a potency compared to rSsp1a at hNa_V_1.7 (Figure 7C; Supplementary Figures S9C).

S7R-E18K-rSsp1a docked at hNa_V_1.2 (Figure 7D; Supplementary Figures S9D) and hNa_V_1.3 (Figure 7E; Supplementary Figures S9E) in a similar way to rSsp1a, maintaining the major S3–S4 loop interactions. However, a new salt bridge was predicted to be formed between the substituted R7 and Glu from the S1–S2 loop in both hNa_V_ subtypes. In addition, the π–π interaction between W24 and Y781 (in hNa_V_1.2) or Y782 (in hNa_V_1.3) potentially stabilized the toxin–channel complex. At hNa_V_1.7, the S7R-E18K-rSsp1a mutant docked in a different orientation to rSsp1a but retained the major R30–E811 and K25–E818 salt bridges, in addition to an extra R7–E759 salt bridge (Figure 7F; Supplementary Figures S9F). The closely oriented W24 and H755 were predicted to form a π–π interaction instead of the W24–E759 electrostatic interaction observed for rSsp1a.

The docking orientation of N14D-P27R-rSsp1a at hNa_V_1.2 and hNa_V_1.7 (Figures 7G, I; Supplementary Figures S9G, I) showed all major salt bridges were retained and the substituted R27 interacted with E785 from the S1–S2 loop in hNa_V_1.2 and the L812 main chain carbonyl oxygen from the S3–S4 loop in hNa_V_1.7. The docking of N14D-P27R-rSsp1a at hNa_V_1.3 closely overlapped the rSsp1a docking orientation, with the loss of a major salt bridge formed by K25 and the substituted R27 did not engage with hNa_V_1.3 DII residues (Figure 7H; Supplementary Figures S9H), contributing to its observed weaker binding at hNa_V_1.3 (Figure 6).

#### 3.3.3 rSsp1a optimization through N-terminal and C-terminal extension

Finally, we extended the rSsp1a N-terminal with GP- to mimic GP-HwTx-IV (Neff et al., 2020) and GP-ProTx-II (Flinspach et al., 2017), while the C-terminal was extended with -GK to mimic the C-terminal amides as previously designed for HwTx-IV (Minassian et al., 2013; Neff et al., 2020). Both GP-rSsp1a and rSsp1a-GK (Supplementary Table S3) did not improve rSsp1a activity or selectivity (Supplementary Figures S5E). Further, the Y26H-rSsp1a mutant with an extra two Leu at the C-terminal (Y26H-rSsp1a-LL) was made to assess the effect of the C-terminal extension with small, linear hydrophobic residues, however, the analogue did not improve the activity or selectivity.

## 4 Discussion

In recent decades, Na_V_-modulating spider ICK toxins have generated significant interest in drug discovery (Dongol et al., 2019), with potential to target Na_V_ subtypes of therapeutic interest (Xie et al., 2015; Su et al., 2017; Salvatierra et al., 2018; Cardoso, 2020; Menezes et al., 2020). Despite the need to discover and develop subtype-selective Na_V_-modulators, few comprehensive structure-function studies of NaSpTxs have been reported, with most focusing on hNa_V_1.7 (Minassian et al., 2013; Revell et al., 2013; Murray et al., 2015; Hu et al., 2021). These findings have helped guide analogue development of NaSpTxs aimed at improving potency and subtype-selectivity for hNa_V_1.7 (Revell et al., 2013; Klint et al., 2015; Murray et al., 2015; Murray et al., 2016; Shcherbatko et al., 2016; Agwa et al., 2017; Flinspach et al., 2017; Rahnama et al., 2017; Zhang et al., 2018; Mueller et al., 2020; Neff et al., 2020; Rupasinghe et al., 2020; Hu et al., 2021), while NaSpTx optimization at other subtypes have been overlooked. This study addresses the gap by investigating the structure-function relationships and optimization of Ssp1a at hNa_V_1.2, hNa_V_1.3 and hNa_V_1.7.

### 4.1 Structure-function of rSsp1a at hNa_V_1.2, hNa_V_1.3 and hNa_V_1.7

The active surface defined for rSsp1a and close homologs GpTx-1 (Murray et al., 2015), HwTx-IV (Revell et al., 2013), and m3-HwTx-IV (Wisedchaisri et al., 2021) are similar, consistent with the concept of conservation in the interaction face of the peptides adopting the same fold with >30% sequence identity (Russell et al., 2004; Tuncbag et al., 2011). These comparisons reveal that the hydrophobic patch of rSsp1a forms the major binding face, with basic residues K25 (loop 4) and R30 (C-terminal) and the hydrophobic Y26 (loop 4) also expected to contribute to binding (Figure 1B; Figures 3A–C). Specifically, the hydrophobic residues W5, F6, W24 and W28 were predicted to be key to the rSsp1a activity. This is consistent with the conservation of Trp, Arg and Tyr in the binding site of peptides/proteins (Moreira et al., 2007), including the NaSpTx1 toxins (Klint et al., 2012; Minassian et al., 2013; Revell et al., 2013; Murray et al., 2015; Wisedchaisri et al., 2021). Although the structure-function of close homologs can be predicted, variations in the selectivity of analogs across Na_V_ subtypes provides an opportunity to optimize peptides to improve potency and selectivity towards a specific target. For example, P27A-rSsp1a only lost activity at hNa_V_1.3, suggesting that the non-conserved Pro at this position likely played less of a structural role in rSsp1a and could be exploited to make designs non-selective to hNa_V_1.3. This is supported by P27R mutation, which significantly improved rSsp1a activity at hNa_V_1.2 while losing activity at hNa_V_1.3 (Figure 4; Supplementary Table S2).

Based on the biophysical studies of rSsp1a (Dongol et al., 2021), we predicted that rSsp1a interacted with hNa_V_ subtypes similarly to HwTx-IV (Xiao et al., 2008) to trap the hNa_V_1.7 DII in the resting state conformation. The available 3D structure of rSsp1a, activity data of rSsp1a alanine mutants, recently published cryo-EM structure of the resting state hNa_V_1.7 DII (Wisedchaisri et al., 2021), previously published channel mutation data (Xiao et al., 2010; Xiao et al., 2011; Cai et al., 2015; Zeng et al., 2018; Xu et al., 2019), and the high sequence homology at the predicted interacting face encouraged data-driven docking of rSsp1a at hNa_V_1.2, hNa_V_1.3 and hNa_V_1.7. Data-driven docking can often accurately predict the molecular interaction at the binding interface in the absence of high-resolution atomic structures to illustrate the molecular interactions (Rodrigues and Bonvin, 2014). The rSsp1a docking across hNa_V_ subtypes revealed the molecular mechanism of interaction is comparable to m3-HwTx-IV–Na_V_1.7 (Wisedchaisri et al., 2021), while highlighting the subtle differences in rSsp1a binding mode across hNa_V_ subtypes (Figures 2, 3), as illustrated by the twisted docking orientation of rSsp1a at hNa_V_1.7 compared to hNa_V_1.2 and hNa_V_1.3. The more hydrophobic LFLA stretch in hNa_V_1.7 DII S3–S4, as compared to LGLA (hNa_V_1.2) and LGLS (hNa_V_1.3), potentially contributed to an observed twist and a shift in the docking of rSsp1a towards the S3–S4 loop in hNa_V_1.7, and to a lesser extent in hNa_V_1.2 and hNa_V_1.3. This shift and twist likely facilitated stronger subtype-specific hydrophobic interactions in hNa_V_1.7, with rSsp1a′s hydrophobic pocket locking the channel’s LFLA stretch, as opposed to hNa_V_1.2 and hNa_V_1.3, where the channel’s hydrophobic patch accommodated the hydrophobic residues of rSsp1a (Figures 3A–C). Additionally, the distinct binding pose of rSsp1a at hNa_V_1.7 also enabled electrostatic interactions with DII S1–S2 loop, which are not observed in hNa_V_1.2 and hNa_V_1.3. Such differences in binding mode across hNa_V_ subtypes provide novel opportunities to optimize toxin-channel interactions across the hNa_V_ subtypes.

The key molecular interactions revealed by the docking studies involve strong salt bridges (≤3 Å) formed between rSsp1a basic residues and the channel subtype’s acidic residues in DII S3–S4 loop (Figures 3A–C), including E753 in the DII S1–S2 loop in hNa_V_1.7 that might allow the rational design of subtype-selective inhibitors. At hNa_V_1.7, additional interactions between indole nitrogen (W24 and W28) and carboxylate (E759 and D816) were observed, possibly strengthening the rSsp1a binding. In contrast, hydrophobic interactions between rSsp1a residue W24 and a Tyr residue in DII S1–S2 loop of hNa_V_1.2 and hNa_V_1.3 were observed. These hydrophobic interactions represent another key molecular mechanism involved in trapping DII in the resting state. For example, prior research (Wisedchaisri et al., 2021) predicted that the hydrophobic interaction between the m3-HwTx-IV hydrophobic patch (I5, F6, W30, and W33) and the LFLA stretch in DII S3–S4 loop (Supplementary Figures S3B) hinders the upward movement of the S4 segment, effectively trapping DII in the resting state. A similar mechanism was observed in the docking of rSsp1a with hNa_V_1.7, although distinct hydrophobic interactions were observed in the case of hNa_V_1.2 and hNa_V_1.3 (Figures 3A–C).

Despite the high sequence homology across Na_V_ subtypes, there is an important variation in the DII S3–S4 hydrophobic stretch in Na_V_1.7 where a bulky hydrophobic Phe replaces the small, non-polar Gly prevalent across other Na_V_ subtypes except Na_V_1.6 and Na_V_1.9. Using the F823G-mNa_V_1.7 variant (equivalent to F813 in hNa_V_1.7) revealed that the Phe unique to hNa_V_1.7 was not critical for rSsp1a interaction (Figure 3D), similarly to HwTx-IV (Xiao et al., 2010). In contrast, this mutation desensitized mNa_V_1.7 binding to Pn3a from NaSpTx2 family by 28-fold (Mueller et al., 2020) and the binding to ProTx-II from NaSpTx3 family by 9–100 fold (Schmalhofer et al., 2008; Xiao et al., 2010). This pharmacology was verified by the structure of ProTx-II–DII-Na_V_Ab complex, where two hydrophobic residues W5 and M6 from ProTx-II flanked F813 to stabilize the DII S3–S4 helix (Xu et al., 2019). However, the docking orientation of rSsp1a (Figure 3C) and m3-HwTx-IV (Supplementary Figures S3B) (Wisedchaisri et al., 2021) at hNa_V_1.7 DII revealed that these two toxins positioned themselves on top of the LFLA motif rather than surrounding F813, agreeing to small reduction of F813G-hNa_V_1.7 or F823G-mNa_V_1.7 affinity to HwTx-IV (Xiao et al., 2010) or rSsp1a, respectively.

### 4.2 Optimization of rSsp1a at hNa_V_1.2, hNa_V_1.3 and hNa_V_1.7

Several optimization studies on NaSpTx toxins have indicated that removing the negative charge at the N-terminal by introducing pyroglutamate, 1-Nal (1-naphthylalanine), Gly, or GP- or simply substituting the acidic residue with Ala or Gly might contribute to toxin binding, potency, and/or selectivity (Minassian et al., 2013; Revell et al., 2013; Rong et al., 2013; Shcherbatko et al., 2016). Extra N-terminal Gly and GP- residues are remnants of TEV and HRV3C digestion of recombinantly expressed fusion proteins, respectively (Klint et al., 2013; Minassian et al., 2013; Flinspach et al., 2017). These non-native residues at the N-terminal are an advantage of the recombinant expression system, as replacing N-terminal acidic residue with Gly in HwTx-IV and HnTx-I has improved the toxin potency (Revell et al., 2013; Rahnama et al., 2017; Zhang et al., 2018). rSsp1a inclusion of a non-native Gly (G0) resulting from TEV digestion and the D1G mutation removed the N-terminal negative charge. In contrast to HwTx-IV (Revell et al., 2013), the D1G-rSsp1a mutant reduced activity at hNa_V_1.7, while no significant changes were observed at hNa_V_1.2 and hNa_V_1.3 (Supplementary Table S2). Instead, a charge reversal mutation (D1K) slightly improved hNa_V_1.2 and hNa_V_1.3 activity with little reduction in hNa_V_1.7 activity, suggesting it could be used when combining mutations to achieve hNa_V_1.2- and/or hNa_V_1.3-selectivity. The D1K mutation is located outside the predicted interacting face of rSsp1a, suggesting this position plays an indirect role in binding, likely through longer-range charge effects. Previous studies have identified that the increased cationicity, as well as the presence of both native and substituted cationic residues (primarily Lys) contributes to the enhanced electrostatic interaction with anionic moieties within the lipid bilayer, including anionic lipid head groups, that can facilitate initial toxin-ion channel interactions (Henriques et al., 2016; Agwa et al., 2017; Agwa et al., 2018; Lawrence et al., 2019). Again, such toxin-membrane lipid binding can be subtype-specific, with D1K-rSsp1a showing reduced potency at hNa_V_1.7 but not at hNa_V_1.2 and hNa_V_1.3.

In rSsp1a loop 1, reinstating cationic residue at position 7 had little effect on rSsp1a potency (Supplementary Figures S5A; Supplementary Table S2) contrasting to the key role played by R7 in GpTx-I activity (Murray et al., 2015; Murray et al., 2016) and K7 in HwTx-IV activity (Minassian et al., 2013) on hNa_V_1.7. Interestingly, reinstating the predominant Lys did not improve rSsp1a potency while restoring the less predominant Arg showed little improvement in the rSsp1a potency, making it a prospective single mutation to combine with other mutations. Further, substituting key residue W5 with less hydrophobic Phe provided rSsp1a hNa_V_1.2-selectivity with <3-fold reduction in potency.

Two mutations in loop 2, N13G and N14D, were anticipated to provide subtype-selectivity and improved potency at hNa_V_1.7, respectively (Minassian et al., 2013; Neff et al., 2020). In contrast to N13G-HwTx-IV (Neff et al., 2020), N13G-rSsp1a lost the activity at hNa_V_1.7. But surprisingly, N14D-rSsp1a significantly lost the hNa_V_1.7 activity with slight improvement in hNa_V_1.2 activity and little reduction in hNa_V_1.3 activity, providing N14D-rSsp1a hNa_V_1.2-selectivity (Figure 6A) and could be considered for combination with other mutations to achieve hNa_V_1.2-selectivity.

Acidic residues in loop 3 are less frequent in NaSpTx1 family toxins (Klint et al., 2012). Therefore, E18 in rSsp1a was substituted to remove the negative charge, reverse the charge, or to introduce hydrophobicity. The charge reversal mutation E18K improved potency at all three hNa_V_ subtypes and its location away from the rSsp1a interaction face, like D1K mutation, suggested its indirect role in potency improvement, as discussed above.

Loop 4 in rSsp1a constitutes active residues, including P27 that was critical only for hNa_V_1.3 activity (Figure 1B). Substituting the rare W24 with more conserved Arg or Ser did not improve rSsp1a activity at hNa_V_ subtypes. W24 was important for rSsp1a activity, contrasting with the equivalent Arg in HwTx-IV and GpTx-1, which was important for HwTx-IV activity at hNa_V_1.2 (Minassian et al., 2013), and GpTx-1 (Murray et al., 2015) and HwTx-IV (Minassian et al., 2013) activity at hNa_V_1.7. Y26H was designed to reinstate more common His, as the equivalent H27A-GpTx-1 decreased in potency at hNa_V_1.7 by >10-fold (Murray et al., 2015; Murray et al., 2016), while D26H-HnTx-I improved hNa_V_1.7 activity by >7-fold (Zhang et al., 2018). However, the design neither improved potency nor selectivity of rSsp1a (Supplementary Figures S5C; Supplementary Table S2), confirming the significance of Y26 as an active residue. The P27R that reinstated the more frequent basic residue in the position improved rSsp1a potency specifically at hNa_V_1.2, suggesting incorporating it in a combined mutation to generate hNa_V_1.2-selective analogues.

The comprehensive substitution of HwTx-IV residues suggested acidic residues at the C-terminal are not preferred to improve activity at hNa_V_1.2 and hNa_V_1.7 (Neff et al., 2020). Thus, D32, which also neighbors the rSsp1a interaction face, was substituted with several functionalities, including polar uncharged, basic, and hydrophobic entities, to evaluate their role in rSsp1a potency and selectivity (Figure 6A; Supplementary Table S2). D32S improved the rSsp1a potency at hNa_V_1.2 (2.5-fold) and hNa_V_1.3 (4-fold) compared to hNa_V_1.7 (1.3-fold) but did not provide subtype-selectivity. In contrast, D32F improved rSsp1a potency only at hNa_V_1.3 (∼3.5-fold) but did not provide hNa_V_1.3-selectivity, given the lower potency of rSsp1a at hNa_V_1.3 compared to remaining two hNa_V_ subtypes. Similarly, the lack of hNa_V_1.3-selectivity was also observed for D32K mutation despite improved hNa_V_1.3 inhibition by 6-fold. Interestingly, D32Y provided hNa_V_1.2-selectivity (5.5-fold) against hNa_V_1.3 with improved potency (3-fold). This contrasts with hNa_V_1.3-active CcoTx-2 which is a natural variant of hNa_V_1.3-inactive CcoTx-1 with D32Y mutation (Bosmans et al., 2006). Thus, D32Y could be combined to generate hNa_V_1.2-selective analogues. Optimization studies of CcoTx-1 (Shcherbatko et al., 2016), GpTx-1 (Murray et al., 2016) and HwTx-IV (Revell et al., 2013; Neff et al., 2020) suggested mutating Y31 in rSsp1a could improve potency and selectivity. Surprisingly, Y31T-rSsp1a did not contribute to hNa_V_1.7-selectivity (Figure 6A), in contrast to the 10-fold hNa_V_1.7-selectivity gained against hNa_V_1.2 by equivalent Y33T-HwTx-IV (Neff et al., 2020). Instead, Y31W-rSsp1a improved the hNa_V_ activity between 1.5 and 3.5-fold, suggesting a preference of bulky hydrophobic in this position.

The potency and selectivity data from single-point mutations guided us to design five double mutants, from which only three designs improved rSsp1a activity (Figures 6A–D). The D1G-D32Y-rSsp1a designed for hNa_V_1.2-selectivity instead improved potency at all three subtypes, showing that the effects of combining single mutations can be unpredictable. Of the 38 optimized rSsp1a analogues, the S7R-E18K-rSsp1a showed the most enhanced potency (up to 10-fold), with the effect of each single mutation being additive when combined and improved potency at all three hNa_V_ subtypes, thus minimising subtype-selectivity (Figure 6A). In contrast to the double mutant S7R-E18K-rSsp1a, the N14D-P27R-rSsp1a double mutant showed additive effects at hNa_V_1.3 but not at hNa_V_1.7, while the neutral effect at hNa_V_1.2 helped it to achieve dual selectivity for hNa_V_1.2 and hNav1.7. While the use of hNa_V_1.2/hNav1.7-selective drugs is typically limited due to potential side effects (Eaton et al., 2021; Zhang et al., 2021; Echevarria-Cooper et al., 2022), this double mutant provides a new starting point for the design of hNa_V_1.2-selective and hNav1.7-selective leads. Overall, rSsp1a optimization by combining two single-point mutations provided two major designs, S7R-E18K with improved potency and N14D-P27R with improved selectivity. Both S7R-E18K and N14D-P27R mutants represent promising starting points for further analogue studies to improve potency and/or subtype-selectivity across hNa_V_1.2, 1.3 and 1.7. Supporting these experimental findings, *in silico* docking studies of optimized rSsp1a analogues revealed key molecular interactions underpinning the improved potency and selectivity observed, including new or altered electrostatic, hydrophobic, π-π interactions, and interactions with the S1–S2 loop of hNa_V_ channels.

In conclusion, we examined the pharmacology of 54 rSsp1a analogues to understand how this NaSpTx1 toxin can be modified to differentially alter interactions at hNa_V_1.2, hNa_V_1.3 and hNa_V_1.7. The inclusion of hNa_V_1.3 interactions provides the first view of NaSpTx1 pharmacology at this subtype. Given Ssp1a is distantly related to HwTx-IV (40% identity), which has comprehensive structure-function and optimization data and shares related pharmacology, the structure-function and optimization data of rSsp1a can be used to enrich the rational design of NaSpTx1 family toxins more broadly. Finally, this study reveals the complexities of moving from single to dual and triple mutations to develop improved research tools and/or potential therapeutic leads.

## Data Availability

The datasets presented in this study can be found in online repositories. The names of the repository/repositories and accession number(s) can be found in the article/Supplementary Material.
