# Supplementary material for: Structure-function and rational design of a spider toxin Ssp1a at human voltage-gated sodium channel subtypes
Source: Front Pharmacol. 2023 Nov 13;14:1277143. doi: 10.3389/fphar.2023.1277143 (PMC10682951; doi:10.3389/fphar.2023.1277143)
Supplement: Supplementary file 1 [file Datasheet1.docx]

Supplementary Material

Structure-function and Rational Design of a Spider Toxin Ssp1a at Human Voltage-gated Sodium Channel Subtypes

Yashad Dongol^1^, David T Wilson^2^, Norelle L Daly^2^, Fernanda C Cardoso^1^, Richard J Lewis^1*^

*** Correspondence:**Richard J Lewis
r.lewis@uq.edu.au

**This file includes:**

Supplementary Figures S1 to S9

Supplementary Tables S1 to S3


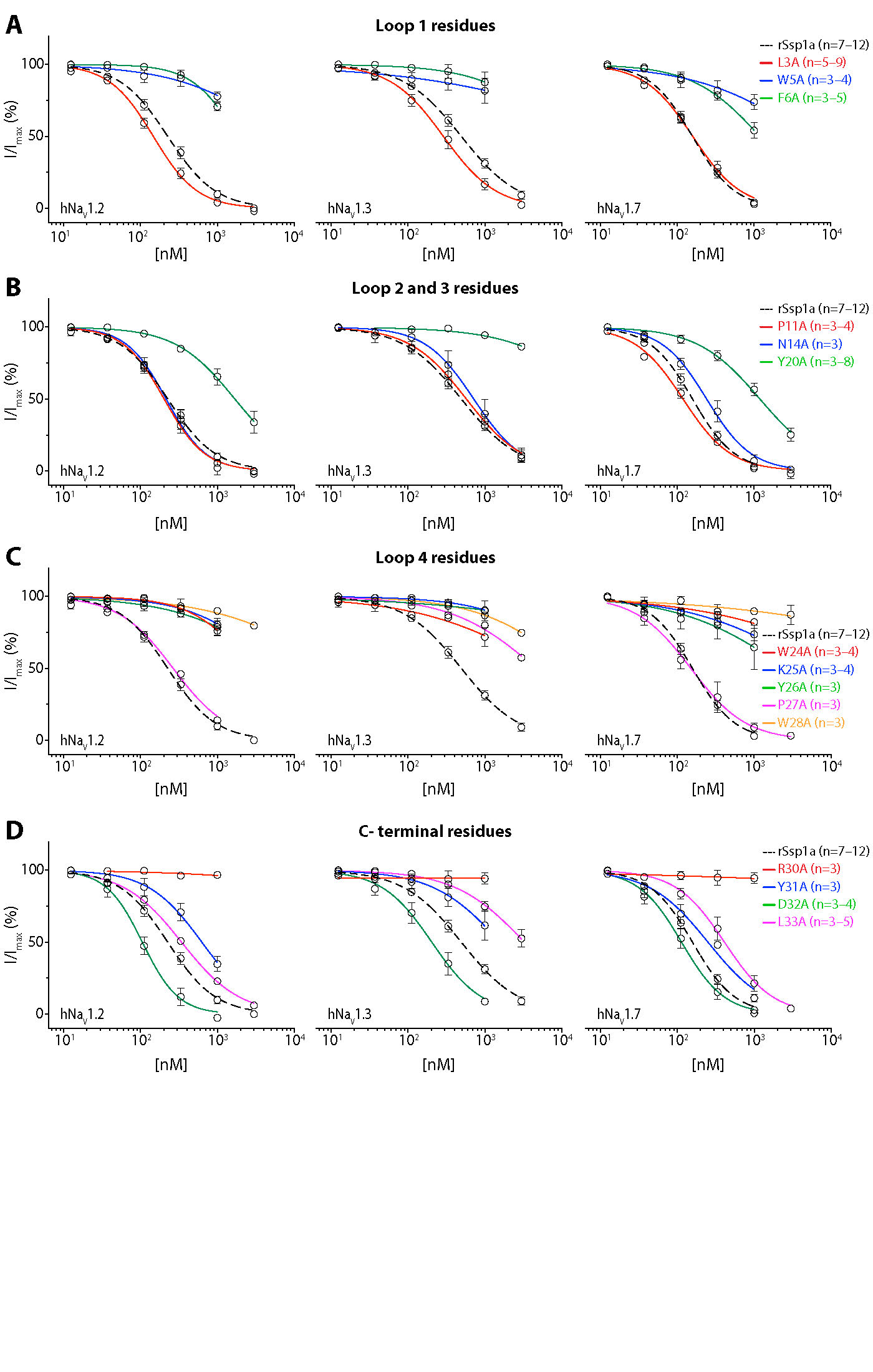


**Supplementary Figure S1. Dose-responses for rSsp1a alanine mutants at hNa_V_1.2, hNa_V_1.3 and hNa_V_1.7. (A to D)** Potency of rSsp1a and alanine mutants were determined via whole-cell automated electrophysiology using QPatch16X and HEK293 cells expressing hNa_V_ subtype with ß1 subunit. Dose-response curves were plotted using data obtained from number of experiments (n) indicated in the legend.


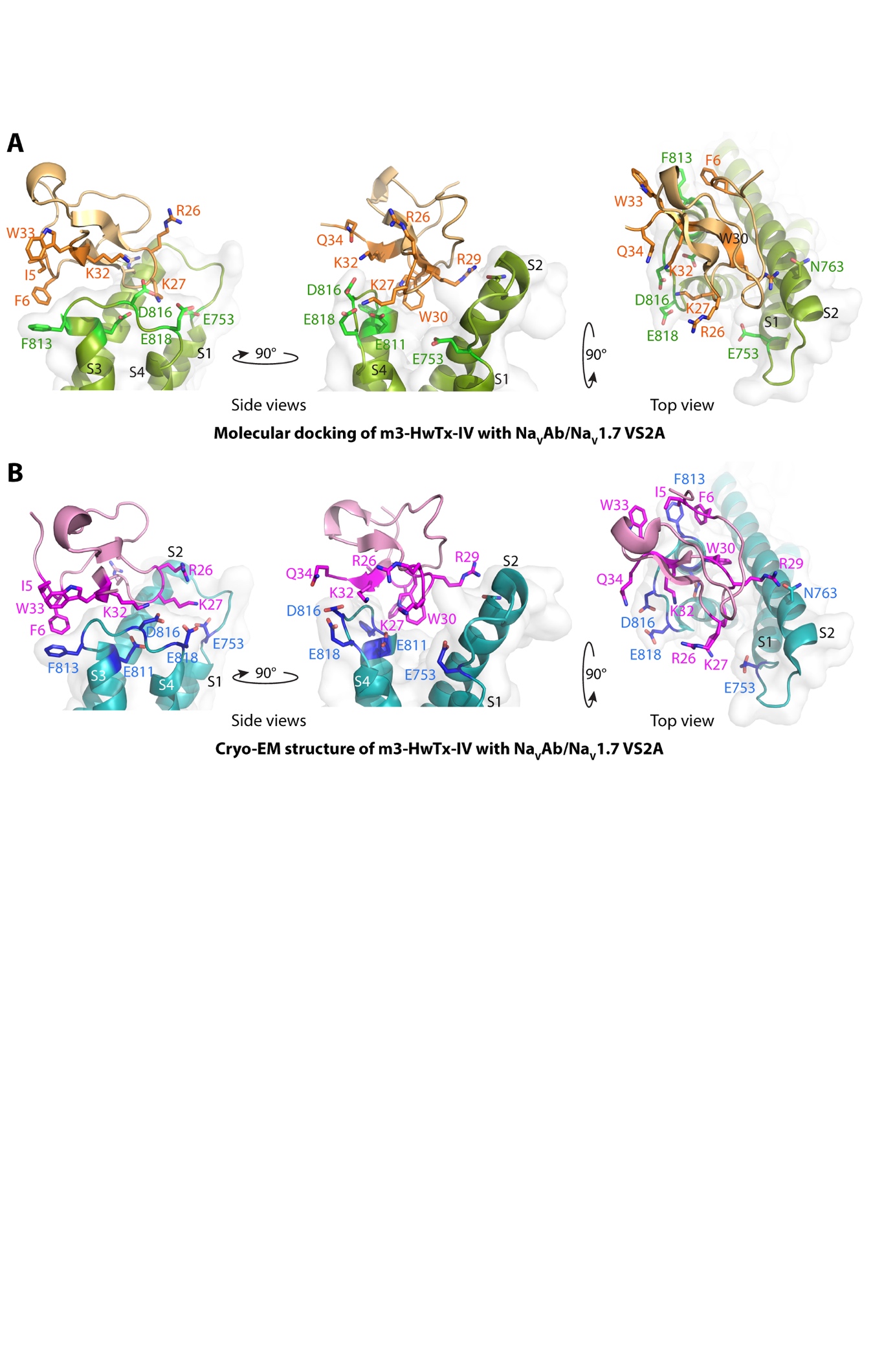


**Supplementary Figure S2. Validating HADDOCK docking by comparing with cryo-EM docking pose of m3-HwTx-IV at Na_V_Ab/Na_V_1.7 VS2A.** **(A)** The m3-HwTx-IV was docked at Na_V_Ab/Na_V_1.7 VS2A using HADDOCK2.2 Easy interface (Van Zundert et al., 2016) and the molecular interactions were compared with **(B)** m3-HwTx-IV–Na_V_Ab/Na_V_1.7 VS2A complex captured by cryo-EM (Wisedchaisri et al., 2021) which revealed similar docking pose of m3-HwTx-IV in both toxin–channel complex, validating HADDOCK docking tool. In both models, key interactions between m3-HwTx-IV and Nav1.7 VS2A were observed, including the electrostatic interactions between negatively charged residues E811, D816 and E818 from DII S3–S4 loop and positively charged K27 and K32, N753 from DII S1–S2 loop and R29, and the hydrophobic interaction between LFLA motif in the DII S3–S4 loop and hydrophobic patch formed by I5, F6, W30 and W33 in the toxin. The root mean square deviation (RMSD) for Cα atoms (123 atoms) between the cryo-EM structure and the HADDOCK model is 0.949 Å.

**
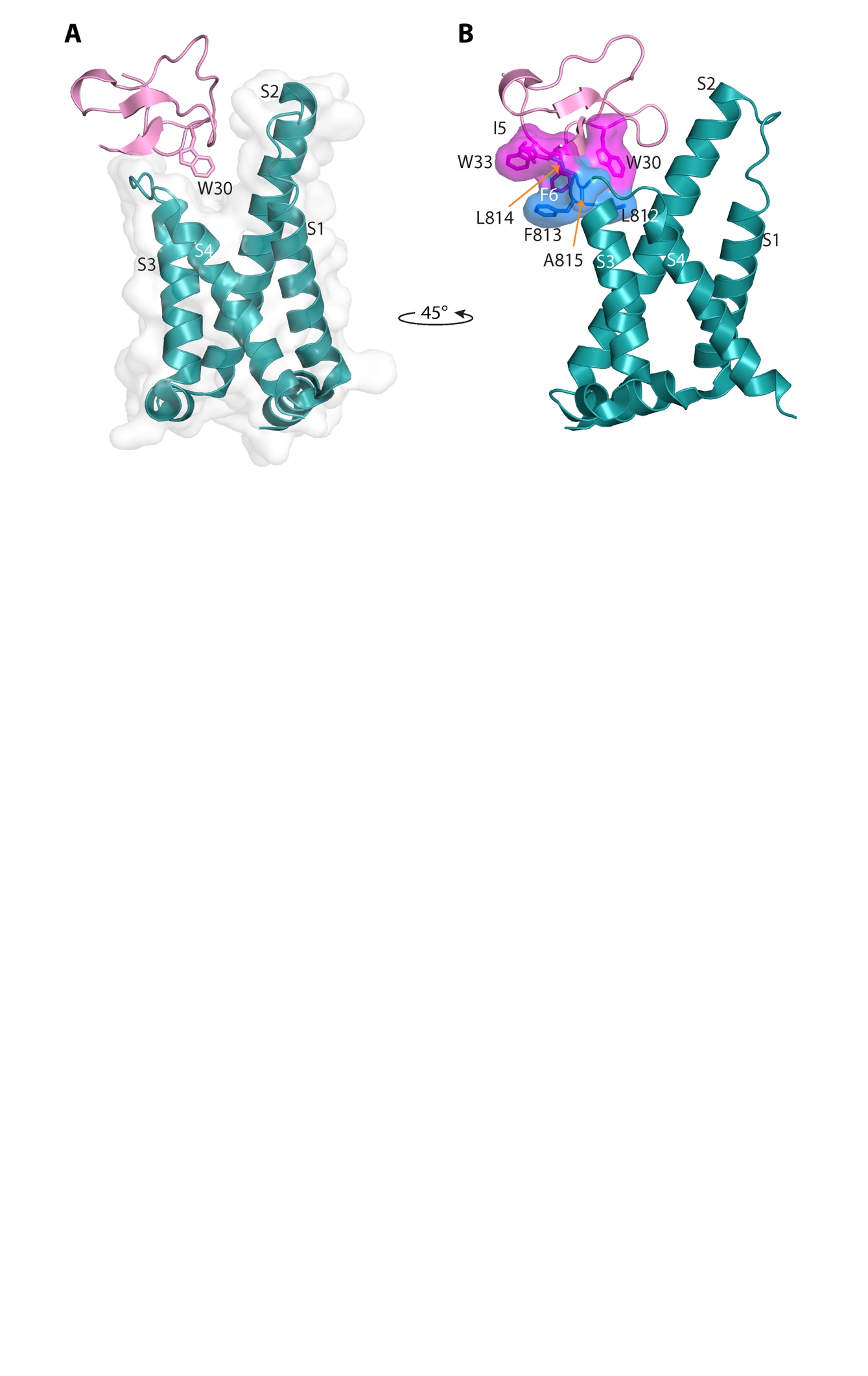
**

**Supplementary Figure S3. Molecular basis of m3-HwTx-IV trapping the DII in resting state in Na_V_Ab/Na_V_1.7 VS2A chimera.** **(A)** m3-HwTx-IV positioned in the aqueous cleft in DII and penetrating deeper in the cleft through W30. **(B)** Hydrophobic patch in m3-HwTx-IV interacting with hydrophobic stretch LFLA in VSD II S3–S4 loop. The cryo-EM structure of m3-HwTx-IV–Na_V_Ab/Na_V_1.7 VS2A complex (Wisedchaisri et al., 2021) was visualized using Pymol 2.4.1 (Schrodinger, 2018) to illustrate the molecular interaction between m3-HwTx-IV and Na_V_Ab/Na_V_1.7 VS2A chimera.

**Supplementary Figure S4.** Key residues in hNa_V_ channel subtypes DII S1–S2 and S3–S4 loop. Key hNa_V_ subtype residues for interaction with inhibitory ICK spider toxins (Xu et al., 2019; Zeng et al., 2018; Cai et al., 2015; Rong et al., 2011; Xiao et al., 2011) are highlighted in boxes and labelled accordingly. Residues predicted by docking studies to be important for rSsp1a binding are shaded in cyan.

**
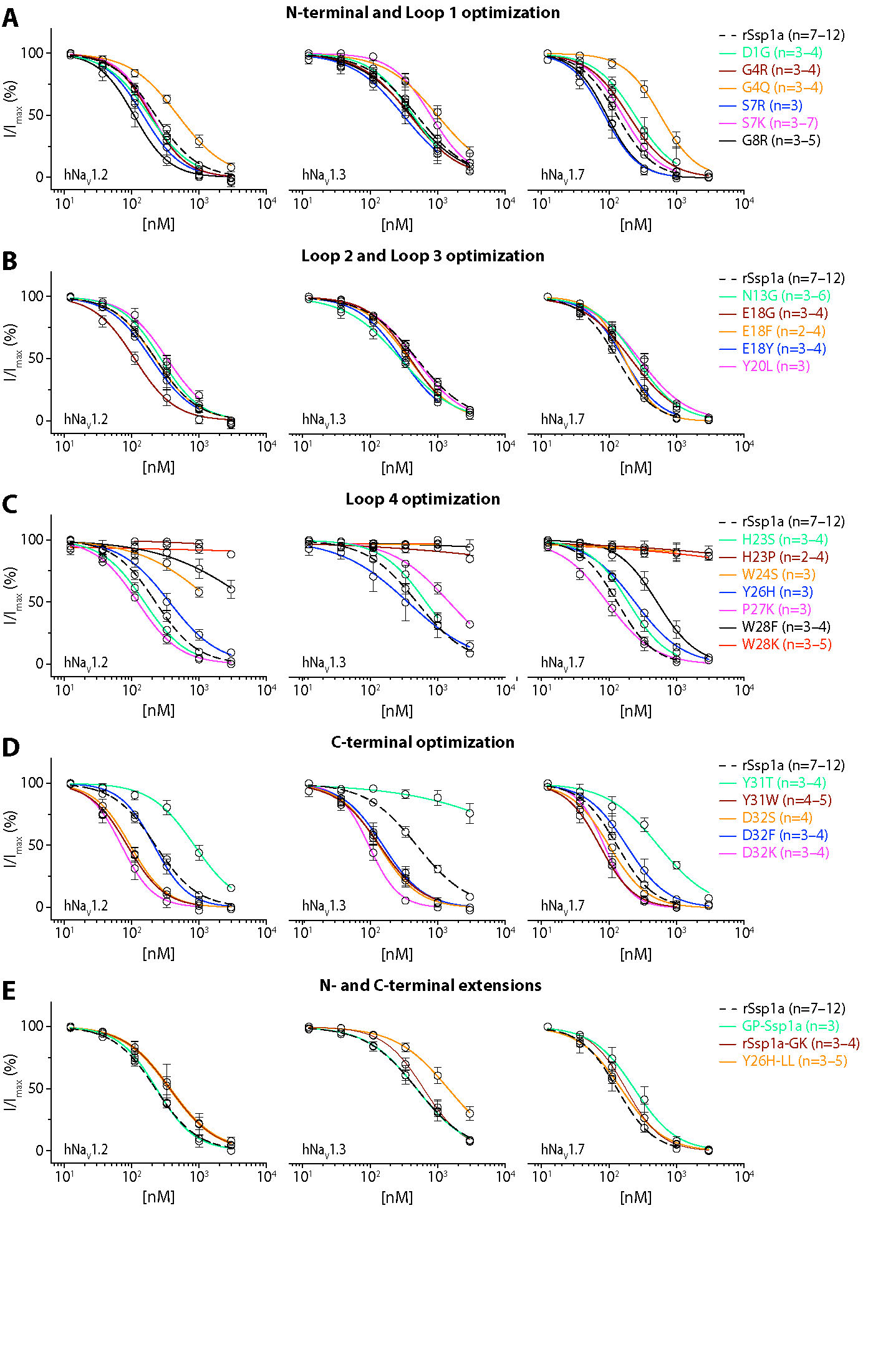
**

**Supplementary Figure S5. Dose-response of optimized rSsp1a analogues. (A to E)** Potency of rSsp1a and analogues were determined via whole-cell automated electrophysiology using QPatch16X and HEK293 cells expressing hNa_V_ subtype with ß1 subunit. Dose-response curves were plotted using data obtained from number of experiments (n) indicated in the legend.

**
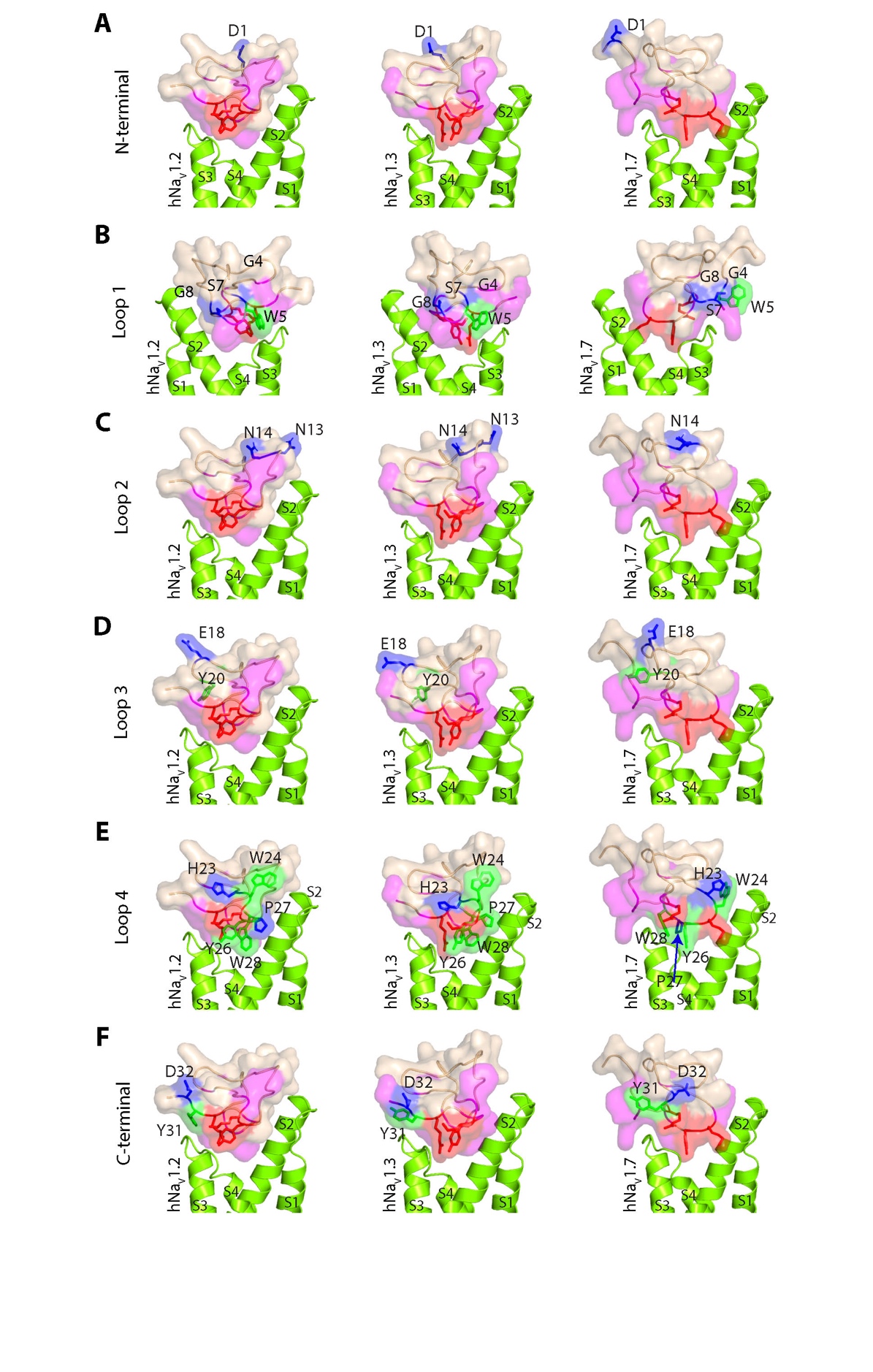
**

**Supplementary Figure S6. Spatial locations of mutated residues. (A to F)** Location of mutated residues (blue) on the rSsp1a surface compared to the rSsp1a interaction face. Residues highlighted in red are key interaction residues (K25, Y26 and R30) whereas magenta indicates the hydrophobic patch on rSsp1a contributing to the rSsp1a interaction face. W5, W28 and Y31 composing hydrophobic patch, buried Y20, and functional residue Y26 that are mutated are highlighted in green.

**
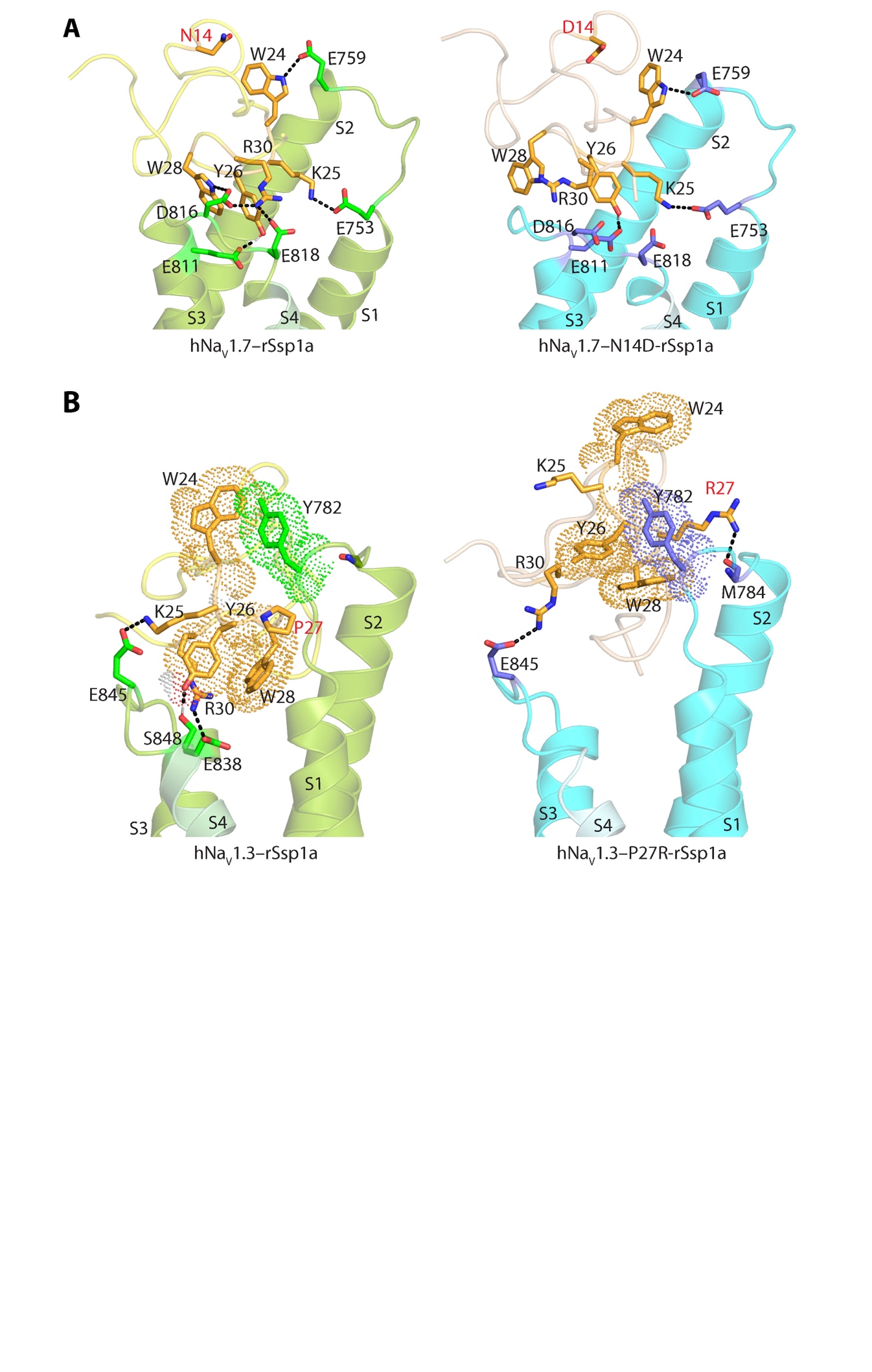
**

**Supplementary Figure S7. Predicted molecular basis for rSsp1a single mutant inactivity. (A)** The docking pose of N14D-rSsp1a at hNa_V_1.7 and **(B)** The docking pose of P27R-rSsp1a at hNa_V_1.3 was chosen in such a way that it closely resembles to rSsp1a docking pose but differs in terms of molecular interaction with the channel to define the probable basis of mutant rSsp1a inactivity at hNa_V_1.7 and at hNa_V_1.3, respectively. The substituted residues are highlighted in red.

**
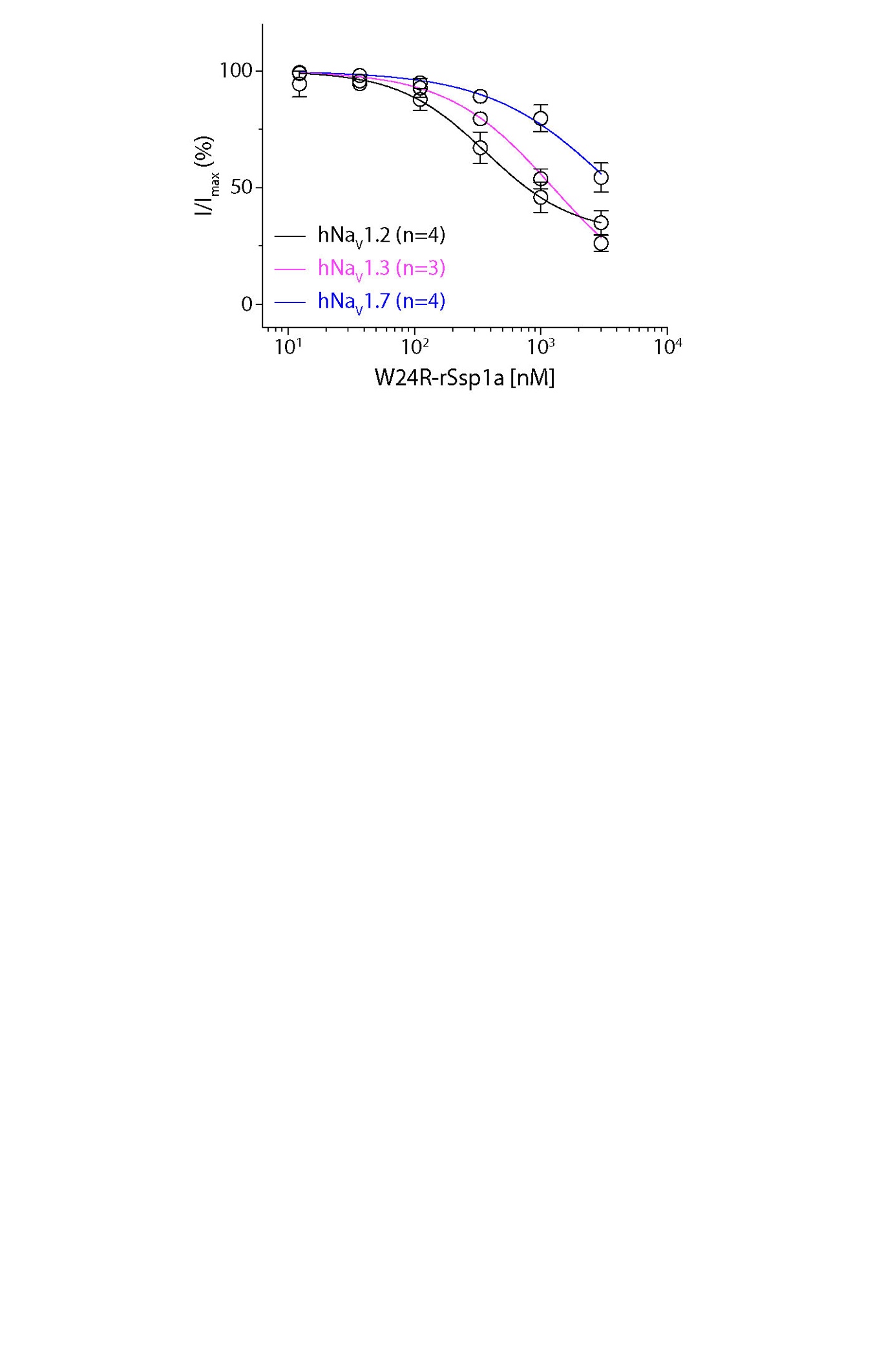
**

**Supplementary Figure S8. Partial inhibition effect of W24R at hNa_V_1.2.** Dose-response curve of rSsp1a analogues compared with rSsp1a at hNa_V_1.2, hNa_V_1.3 and hNa_V_1.7, obtained using whole-cell automated patch clamp electrophysiology (QPatch 16X) platform. Dose-response curves were plotted using data obtained from number of experiments (n) indicated in the legend.

**
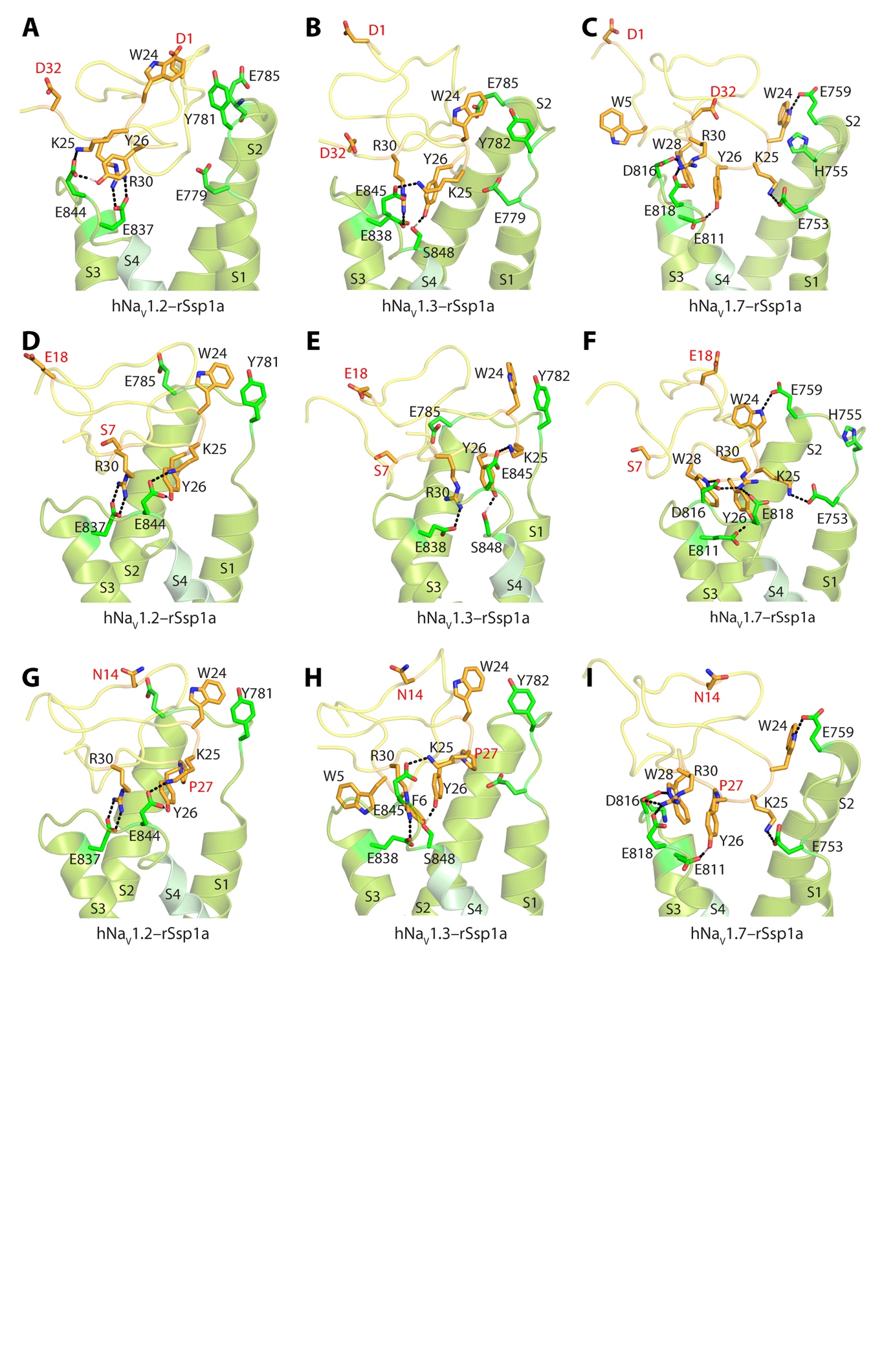
**

**Supplementary Figure S9. rSsp1a docking controls for double mutant docking in Figure 7. (A to I)** rSsp1a docking pose aligned to the rSsp1a double mutant docking pose illustrated in Figure 7 to visualize the molecular interaction of rSsp1a and rSsp1a double mutants at hNa_V_1.2, hNa_V_1.3 and hNa_V_1.7.

**Supplementary Table S1. Mean IC_50_ of rSsp1a alanine mutants at hNa_V_1.2, hNa_V_1.3 and hNa_V_1.7.** Data were presented as means ± SEM, with number of experiments (n) indicated. The potency of each mutant is compared with rSsp1a potency at a given hNa_V_ subtype. *, **, ***, and **** refer *p* values at ≤0.05, ≤0.01, ≤0.001, and ≤0.000, respectively.

| SNo | rSsp1a analogues | hNa_V_1.2 | | | hNa_V_1.3 | | | hNa_V_1.7 | | |
| --- | --- | --- | --- | --- | --- | --- | --- | --- | --- | --- |
|  |  | Mean IC_50_ (nM) | SEM | n | Mean IC_50_ (nM) | SEM | n | Mean IC_50_ (nM) | SEM | n |
| **1** | **rSsp1a** | **239** | **26** | **7** | **547** | **70** | **12** | **134** | **9** | **7** |
| 2 | L3A | 154* | 19 | 7 | 311 | 59 | 5 | 170 | 20 | 9 |
| 3 | W5A | >1000 |  | 4 | >1000 |  | 3 | >1000 |  | 3 |
| 4 | F6A | >1000 |  | 3 | >1000 |  | 3 | >1000 |  | 5 |
| 5 | P11A | 208 | 26 | 4 | 600 | 136 | 3 | 120 | 8 | 4 |
| 6 | N14A | 224 | 42 | 3 | 784 | 215 | 3 | 252** | 52 | 3 |
| 7 | Y20A | 1842*** | 430 | 3 | >3000 |  | 8 | 1275**** | 208 | 6 |
| 8 | W24A | >1000 |  | 3 | >1000 |  | 3 | >1000 |  | 3 |
| 9 | K25A | >1000 |  | 3 | >1000 |  | 3 | >1000 |  | 4 |
| 10 | Y26A | >1000 |  | 3 | >1000 |  | 3 | >1000 |  | 3 |
| 11 | P27A | 270 | 37 | 3 | >3000 |  | 3 | 166 | 48 | 3 |
| 12 | W28A | >3000 |  | 3 | >3000 |  | 3 | >3000 |  | 3 |
| 13 | R30A | >1000 |  | 3 | >1000 |  | 3 | >1000 |  | 3 |
| 14 | Y31A | 633** | 120 | 3 | >1000 |  | 3 | 254** | 41 | 3 |
| 15 | D32A | 110* | 21 | 3 | 221* | 49 | 4 | 118 | 24 | 3 |
| 16 | L33A | 396 | 141 | 3 | >3000 |  | 4 | 448** | 103 | 5 |

**Supplementary Table S2. Mean IC_50_ of rSsp1a non-alanine mutants at hNa_V_1.2, hNa_V_1.3 and hNa_V_1.7.** Data were presented as means ± SEM, with number of experiments (n) indicated. The potency of each mutant is compared with rSsp1a potency at a given hNa_V_ subtype. *, **, ***, and **** refer *p* values at ≤0.05, ≤0.01, ≤0.001, and ≤0.000, respectively.

| SN | rSsp1a analogues | hNa_V_1.2 | | | hNa_V_1.3 | | | hNa_V_1.7 | | |
| --- | --- | --- | --- | --- | --- | --- | --- | --- | --- | --- |
|  |  | Mean IC_50_  (nM) | SEM | n | Mean IC_50_  (nM) | SEM | n | Mean IC_50_  (nM) | SEM | n |
| **1** | **rSsp1a** | **239** | **26** | **7** | **547** | **70** | **12** | **134** | **9** | **7** |
| 2 | D1G | 192 | 33 | 4 | 499 | 146 | 3 | 307* | 121 | 3 |
| 3 | D1K | 97** | 11 | 4 | 196* | 25 | 3 | 240 | 71 | 4 |
| 4 | G4R | 193 | 15 | 4 | 409 | 72 | 3 | 201* | 30 | 3 |
| 5 | G4Q | 503** | 70 | 4 | 999* | 255 | 4 | 606*** | 113 | 4 |
| 6 | W5F | 579** | 151 | 3 | >3000 |  | 3 | 1260** | 423 | 3 |
| 7 | S7R | 153 | 19 | 3 | 333 | 78 | 3 | 94 | 20 | 3 |
| 8 | S7K | 201 | 17 | 3 | 929 | 337 | 3 | 181 | 28 | 7 |
| 9 | G8R | 117** | 25 | 5 | 415 | 44 | 3 | 96* | 12 | 4 |
| 10 | N13G | 280 | 45 | 3 | 287 | 37 | 4 | 287* | 53 | 6 |
| 11 | N14D | 126* | 9 | 4 | 900 | 124 | 4 | >3000 |  | 3 |
| 12 | E18G | 119* | 23 | 4 | 406 | 59 | 3 | 276 | 96 | 4 |
| 13 | E18F | 232 | 51 | 2 | 395 | 61 | 4 | 171 | 13 | 3 |
| 14 | E18Y | 203 | 28 | 3 | 300 | 35 | 3 | 179 | 35 | 4 |
| 15 | E18K | 72** | 5 | 3 | 313 | 82 | 4 | 107 | 15 | 3 |
| 16 | Y20L | 340 | 40 | 3 | 476 | 60 | 3 | 288** | 58 | 3 |
| 17 | H23S | 156 | 16 | 4 | 679 | 111 | 3 | 198* | 23 | 3 |
| 18 | H23P | >3000 |  | 2 | >3000 |  | 4 | >3000 |  | 3 |
| 19 | W24S | 1456**** | 188 | 3 | >3000 |  | 3 | >3000 |  | 3 |
| 20 | W24R | 423* | 81 | 4 | 1185** | 153 | 3 | >3000 |  | 4 |
| 21 | Y26H | 369 | 72 | 3 | 484 | 282 | 3 | 244*** | 16 | 3 |
| 22 | Y26H-LL | 410 | 90 | 5 | 1626*** | 328 | 5 | 175 | 60 | 3 |
| 23 | P27R | 77** | 5 | 4 | >1000 |  | 3 | 141 | 22 | 3 |
| 24 | P27K | 124* | 10 | 3 | 1525**** | 193 | 3 | 108 | 23 | 3 |
| 25 | W28F | >3000 |  | 4 | >3000 |  | 3 | 544*** | 116 | 3 |
| 26 | W28K | >3000 |  | 3 | >3000 |  | 3 | >3000 |  | 4 |
| 27 | Y31T | 874**** | 126 | 3 | >3000 |  | 3 | 545*** | 106 | 4 |
| 28 | Y31W | 89** | 13 | 4 | 160** | 38 | 5 | 80* | 19 | 4 |
| 29 | D32F | 219 | 24 | 3 | 162* | 29 | 3 | 203 | 48 | 4 |
| 30 | D32S | 97** | 12 | 4 | 137** | 18 | 4 | 101* | 6 | 4 |
| 31 | D32K | 75** | 17 | 3 | 98** | 12 | 3 | 86** | 5 | 4 |
| 32 | D32Y | 75** | 8 | 3 | 417 | 84 | 3 | 110 | 11 | 4 |
| 33 | Pro-Ssp1a | 239 | 26 | 3 | 533 | 126 | 3 | 259* | 69 | 3 |
| 34 | rSsp1a-GK | 426 | 136 | 3 | 654 | 129 | 4 | 180* | 19 | 3 |
| 35 | D1G/D32Y | 86** | 5 | 4 | 111** | 16 | 4 | 91** | 7 | 4 |
| 36 | E18K/S7R | 40*** | 2 | 4 | 59** | 13 | 3 | 40**** | 3 | 4 |
| 37 | N14D/P27R | 87** | 3 | 3 | >3000 |  | 3 | 57*** | 3 | 3 |
| 38 | W5F/N14D | 735*** | 102 | 3 | 1202** | 207 | 3 | 391** | 118 | 3 |
| 39 | S7K/W28F | >3000 |  | 3 | >3000 |  | 3 | 1472**** | 156 | 4 |

**Supplementary Table S3. Primary structure of N- and C-terminal extended rSsp1a** **analogues.**

|  | **Peptide** | **Sequence** |
| --- | --- | --- |
| 1 | rSsp1a | ^0^G-DCLGWFSGCDPNNNKCCEGYVCHWKYPWCRYDL^33^ |
| 2 | GP-Ssp1a | ^0^GPDCLGWFSGCDPNNNKCCEGYVCHWKYPWCRYDL^34^ |
| 3 | rSsp1a-GK | ^0^G-DCLGWFSGCDPNNNKCCEGYVCHWKYPWCRYDLGK^35^ |
| 4 | Y26H-rSsp1a-LL | ^0^G-DCLGWFSGCDPNNNKCCEGYVCHWKHPWCRYDLLL^35^ |

**Uncategorized References**

Cai, T., Luo, J., Meng, E., Ding, J., Liang, S., Wang, S., et al. (2015). Mapping the interaction site for the tarantula toxin Hainantoxin-IV (β-TRTX-Hn2a) in the voltage sensor module of domain II of voltage-gated sodium channels. *Peptides* 68: 148-56. <https://doi.org/10.1016/j.peptides.2014.09.005>.

Rong, M., Chen, J., Tao, H., Wu, Y., Jiang, P., Lu, M., et al. (2011). Molecular basis of the tarantula toxin Jingzhaotoxin-III (β-TRTX-Cj1α) interacting with voltage sensors in sodium channel subtype Na_V_1.5. *FASEB J.* 25: 3177-3185. <https://doi.org/10.1096/fj.10-178848>.

Schrodinger. (2018). The PyMOL Molecular Graphics System, Version 2.0.

Van Zundert, G., Rodrigues, J., Trellet, M., Schmitz, C., Kastritis, P., Karaca, E., et al. (2016). The HADDOCK2.2 web server: user-friendly integrative modeling of biomolecular complexes. *J. Mol. Biol.* 428: 720-725. <https://doi.org/10.1016/j.jmb.2015.09.014>.

Wisedchaisri, G., Tonggu, L., Gamal El-Din, T. M., McCord, E., Zheng, N., and Catterall, W. A. (2021). Structural basis for high-affinity trapping of the Na_V_1.7 channel in its resting state by tarantula toxin. *Mol. Cell* 81: 38-48.e4. <https://doi.org/10.1016/j.molcel.2020.10.039>.

Xiao, Y., Jackson, J. O., Liang, S., and Cummins, T. R. (2011). Common molecular determinants of tarantula Huwentoxin-IV inhibition of Na^+^ channel voltage-sensors in domains II and IV. *J. Biol. Chem.* 286: 27301-27310. <https://doi.org/10.1074/jbc.M708447200>.

Xu, H., Li, T., Rohou, A., Arthur, C. P., Tzakoniati, F., Wong, E., et al. (2019). Structural basis of Na_V_1.7 inhibition by a gating-modifier spider toxin. *Cell* 176: 702-715. <https://doi.org/10.1016/j.cell.2018.12.018>.

Zeng, X., Li, P., Chen, B., Huang, J., Lai, R., Liu, J., et al. (2018). Selective closed-state Na_V_1.7 blocker JzTx-34 exhibits analgesic effects against pain. *Toxins (Basel)* 10: E64. <https://doi.org/10.3390/toxins10020064>.
